# The future of brain tumor treatment: integrating platelet-based liquid biopsy, advanced drug delivery systems, and eicosanoid inhibition

**DOI:** 10.3389/fphar.2026.1956969

**Published:** 2026-10-01

**Authors:** Sara Di Berardino, Marta Zarà, Huzaifa Ali Ahfaz, Stefania Tacconelli, Paola Patrignani

**Affiliations:** 1 Systems Pharmacology and Translational Therapeutics Laboratory, at the Center for Advanced Studies and Technology (CAST), and Department of Neuroscience, Imaging and Clinical Science, “G. d'Annunzio” University, Chieti, Italy; 2 Department of Biology and Biotechnology, University of Pavia, Pavia, Italy; 3 IRCCS Policlinico San Matteo Foundation, Pavia, Italy

**Keywords:** brain tumors, drug delivery, eicosanoids, liquid biopsy, platelets, tumor-educated platelets

## Abstract

Despite extensive research, brain tumors still have some of the lowest survival rates among cancers. Their marked resistance to both conventional and emerging therapies stems from the unique properties of neural cells and their microenvironment, including the blood-brain barrier, cellular heterogeneity, and an immunosuppressive milieu. Addressing these challenges is essential for developing effective therapeutic strategies that improve patient outcomes. Advancements in brain tumor treatment depend on understanding the biological mechanisms behind potential drug targets and developing appropriate drug discovery strategies to inhibit them effectively. A major barrier remains the difficulty many drugs face in crossing the blood-brain barrier, which greatly restrains treatment effectiveness in brain tumor patients. Therefore, developing innovative drug delivery systems is essential. The brain exhibits a high concentration of fatty acids, notably polyunsaturated fatty acids such as arachidonic acid (AA), which are primarily esterified within membrane phospholipids. Upon release, cyclooxygenases, lipoxygenases, and cytochrome P450 enzymes can transform AA into various eicosanoid classes, producing lipid mediators involved in signal transduction pathways that regulate neurotransmission, neuroinflammation, and neuroplasticity. The diverse eicosanoid families can be synthesized within brain tumors and may contribute to tumor heterogeneity. This includes cell-specific eicosanoid production involving cancer cells, microglia, infiltrating immune cells, and platelets, each expressing distinct enzymes that facilitate tumor progression and immune evasion. This review examines the various mechanisms underlying brain cancer biology, with a particular focus on the roles of eicosanoids and their signaling pathways and how they affect tumor development, progression, and resistance to therapies. The review also describes therapeutic strategies targeting these pathways. The challenge of reduced responsiveness to therapeutic interventions is addressed through platelet-based drug delivery systems, which can increase efficacy by transporting chemotherapeutic agents directly to cancer cells. These systems also include nanoparticles coated with platelet membranes to evade immune clearance of intact cells and extracellular vesicles that provide high biocompatibility. Finally, tumor-educated platelets—which carry RNA and protein cargo—are presented as noninvasive biomarkers for diagnosing and monitoring disease progression and response to therapies in brain tumors.

## 1 Introduction

Brain tumors account for approximately 80%–95% of all central nervous system (CNS) neoplasms, which also include the less common spinal cord tumors. Despite their relatively infrequent occurrence compared to other cancer types, they present a substantial global health concern due to their aggressive behavior, poor prognosis, and significant impact on neurological functions (Ostrom et al., 2023). These tumors involve both glial and non-glial tissues. The RARECARE project estimates the burden—encompassing incidence, prevalence, survival rates, and the proportion of cured cases—of primary CNS cancers within the European Union (EU 27, including 27 member states) using population-based cancer registry data (Crocetti et al., 2012). The most common tumors were astrocytic, accounting for 86%, with 24% classified as low-grade (WHO Grade 1 or 2), 63% as high-grade, fast-growing, and highly malignant (WHO Grade 3 or 4, such as glioblastoma [GBM]), and 13% as gliomas not specified (Crocetti et al., 2012). Additional tumor types included oligodendroglial (4%), ependymal (3.6%), embryonal (4.1%), and choroid plexus carcinomas (0.1%). Astrocytic tumors have an estimated annual incidence of about 3–5 cases per 100,000 individuals, significantly lower than other cancer types. For example, breast cancer and prostate cancer can have annual incidence rates of 100 cases per 100,000 individuals. Five-year relative survival ranged from 4.9% in high-grade astrocytic tumors to over 54% in oligodendroglial tumors (Crocetti et al., 2012). These numbers are much lower than survival rates for both breast cancer and prostate cancer, which approach nearly 100% when detected early but vary widely depending on the stage at diagnosis. This indicates that early detection is essential for enhancing cancer prognosis and survival rates. Early detection of brain cancer is difficult because reliable, non-invasive biomarkers—biological indicators in blood or other body fluids—are often absent or hard to identify at an early stage (Wasiak et al., 2026). Addressing this critical medical need is urgent. Another crucial aspect related to the failure of brain cancer treatment is the marked resistance observed against both conventional and emerging therapeutic strategies. This resistance involves several complex factors, including the restrictive nature of the blood-brain barrier (BBB), significant cellular heterogeneity within tumors, and an immunosuppressive microenvironment (Miyazaki et al., 2020).

This review underlines that simultaneous advances in innovative pharmacological therapies, brain delivery methods, and noninvasive biomarkers can address current challenges in brain cancer treatment, ultimately improving patient outcomes and survival rates.

The brain exhibits a high concentration of fatty acids (FAs), notably polyunsaturated fatty acids (PUFAs) such as arachidonic acid (AA), which are primarily esterified within membrane phospholipids. Upon release, cyclooxygenases, lipoxygenases, and cytochrome P450 enzymes can transform AA into various eicosanoid classes, producing lipid mediators involved in signal transduction pathways that regulate neurotransmission, neuroinflammation, and neuroplasticity. This review discusses the roles of eicosanoids and their influence on tumor growth, progression, and resistance to therapies. It also underlines the development of platelet-based drug delivery systems to promote the transport of chemotherapeutic agents to cancer cells. These systems include nanoparticles coated with platelet membranes to avoid immune clearance of intact cells, as well as extracellular vesicles (EVs) that offer high biocompatibility. Additionally, the review reports how tumor-educated platelets containing RNA and protein cargo can act as noninvasive biomarkers for diagnosing and monitoring disease progression and therapy response in brain tumors.

## 2 The oxylipins

FAs are classified by the number of double bonds in their side chains: saturated FAs (SFAs) have none; monounsaturated FAs (MUFAs) have one; and polyunsaturated FAs (PUFAs) have two or more (Burr and Burr, 1930). FAs are also distinguished by their chain length and the position of their first double bond at the methyl end, known as omega (ω) or n−FAs. For instance, all-cis-9,12,15-octadecatrienoic acid (α-linolenic acid, ALA, C18:3) and all-cis-9,12-octadecadienoic acid (linoleic acid, LA, C18:2) are classified as n−3 FA and n−6 FA, respectively, and are essential FAs because the human body cannot produce them and they must be obtained through diet (Sprecher, 2000). Long-chain (LC) FAs have more than 12 carbons, and those with 22 or more are called very-long-chain (VLC) FAs. LC-PUFAs like LA and ALA are converted enzymatically *in vivo* into VLC-PUFAs (Nakamura and Nara, 2004). Notably, LC- and VLC-PUFAs include important compounds such as all-cis-5,8,11,14,17-eicosapentaenoic acid (EPA, 20:5, n−3), all-cis-7,10,13,16,19-docosapentaenoic acid (DPA, C22:5, n−3), all-cis-4,7,10,13,16,19-docosahexaenoic acid (DHA, C22:6, n−3), and all-cis-5,8,11,14-eicosatetraenoic acid (AA, C20:4, n−6), which are important for various physiological functions (Calder, 2012) (Table 1). In the brain, PUFAs regulate both the structure and the function of neurons, glial cells, and endothelial cells (Bazinet and Layé, 2014). Recently, mechanisms by which PUFAs enter and are regulated within the brain have been identified and characterized; studies have shown crucial roles for PUFAs in neuronal survival, neurogenesis, synaptic function, and the regulation of brain inflammation (Tian et al., 2025). Oxylipins are oxygenated compounds derived from FAs, formed through mono- or dioxygenase enzyme processes (O’Donnell, 2025). Among these, eicosanoids—though often referred to by this name despite the preferred IUPAC term ‘icosanoid’—are metabolites from 20-carbon PUFAs. They are produced via enzymatic pathways mainly involving three oxygenase enzyme families: cyclooxygenases (COXs), P450 cytochrome epoxygenases (CYP450), and lipoxygenases (LOXs) (Needleman et al., 1986). Eicosanoids include prostaglandins (PGs) and thromboxane A_2_ (TXA_2_), collectively called prostanoids, as well as leukotrienes (LTs), hydroperoxyeicosatetraenoic acids (HpETEs), hydroxyeicosatetraenoic acids (HETEs), and oxo-eicosatetraenoic acids (oxo-ETEs) (Samuelsson, 1983). These are generated by various dehydrogenases acting on HETEs or by dehydration of HpETEs (Powell and Rokach, 2015) (Figure 1). Recent reports have identified specialized pro-resolving mediators (SPMs), including lipoxins (LXs), resolvins, protectins (PDs), their aspirin-triggered isomers, and maresins (MaRs). LXs are derived from AA, E-series resolvins from EPA, and D-series resolvins, PDs, and MaRs from DHA. These mediators are suggested to play key roles in resolving inflammation, a hypothesis supported by the availability of synthetic standards and pharmacological studies (Parkinson, 2006). However, doubts remain about whether SPMs are naturally occurring mediators. Additionally, many studies lack reliable evidence for SPM presence in biological samples because of substandard analytical methods or nonspecific assays, and artifacts from PUFA autoxidation may be misinterpreted as SPMs (O’Donnell et al., 2023; Schebb et al., 2022). This review does not describe SPMs; it focuses on the biosynthesis of eicosanoids derived from AA obtained through the action of COXs, LOXs, and CYP450s.

**TABLE 1:** Classification of selected polyunsaturated fatty acids according to chain length and omega family.

Fatty acid	Abbreviation	Notation	Class	Omega family
α-Linolenic acid	ALA	C18:3	LC-PUFA (C18 PUFA)	*n*-3
Linoleic acid	LA	C18:2	LC-PUFA (C18 PUFA)	*n*-6
Eicosapentaenoic acid	EPA	C20:5	LC-PUFA	*n*-3
Arachidonic acid	AA	C20:4	LC-PUFA	*n*-6
Docosapentaenoic acid	DPA	C22:5	VLC-PUFA	*n*-3
Docosahexaenoic acid	DHA	C22:6	VLC-PUFA	*n*-3

Abbreviations: α-Linolenic acid (ALA), linoleic acid (LA), eicosapentaenoic acid (EPA), arachidonic acid (AA), docosapentaenoic acid (DPA), and docosahexaenoic acid (DHA); PUFA, Polyunsaturated fatty acid; LC-PUFA, Long-chain polyunsaturated fatty acid; VLC-PUFA, Very-long-chain polyunsaturated fatty acid.

**FIGURE 1:**
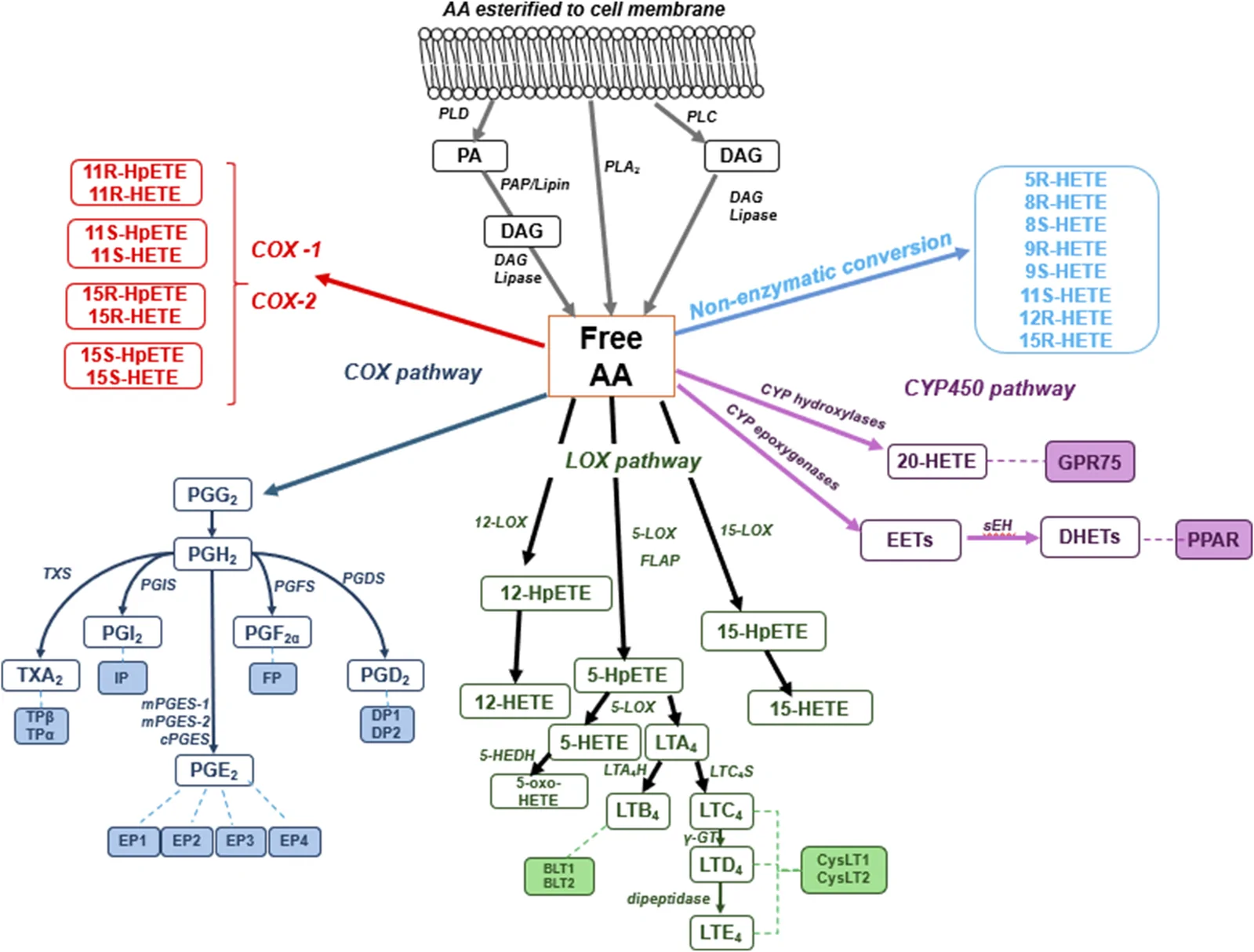
Overview of arachidonic acid metabolism through enzymatic and non-enzymatic pathways. Arachidonic acid (AA) is released from its esterified position at the sn-2 site of phospholipids (PL) in cell membranes primarily by the activity of cytosolic phospholipase A_2_ (cPLA_2_); phospholipase C (PLC) and phospholipase D (PLD) can also contribute to the release of AA but indirectly, by producing diacylglycerol (DAG) and phosphatidic acid (PA), respectively. PA is then metabolized to DAG by phosphatidate phosphohydrolase (PAP), also known as lipin; DAG lipase acts on DAG to release free AA. Free AA is metabolized by cyclooxygenase (COX, shown in blue), lipoxygenase (LOX, shown in green), and cytochrome P450 (CYP450, shown in purple) pathways. Additionally, auto-oxidation of AA can generate multiple positional and stereochemical HETEs (hydroxyeicosatetraenoic acids) (shown in light blue). COX-1/2 convert AA to prostaglandin (PG)G_2_ and then to PGH_2_ by cyclooxygenase and peroxidase activities, respectively; PGH_2_ is then converted by downstream prostanoid synthases, also called terminal prostaglandin synthases or tissue-specific isomerases that act as isomerases that convert unstable PGH2 into specific bioactive prostanoids: thromboxane (TX)A_2_, prostacyclin (PGI_2_), PGE_2_, PGD_2_ and PGF_2_α, which signal through their corresponding specific prostanoid receptors. COX-1/2 convert AA to HETEs as minor products (shown in red). In the LOX pathway, 5-, 12-, and 15-LOX enzymes convert AA to hydroperoxyeicosatetraenoc acids (HpETEs), which are reduced to respective HETEs. The 5-HpETE is metabolized by 5-LOX to generate leukotriene (LT) A4, which is further converted enzymatically into LTB_4_ or the cysteinyl leukotrienes LTC_4_/LTD_4_/LTE_4_. LTB_4_ and LTD_4_ act via specific receptor activation. Two distinct pathways are active in the cytochrome P450 (CYP 450) pathway, where CYP hydroxylases generate 20-HETE, which signals through the GPR75 receptor, and CYP epoxygenases generate epoxyeicosatrienoic acids (EETs), which can engage PPAR signaling. EETs can also be converted to dihydroxyeicosatrienoic acids (DHETs) through soluble epoxide hydrolase (sEH). Solid arrows indicate enzymatic reactions, dashed lines indicate receptor binding or signaling, and solid lines indicate non-enzymatic conversion.

### 2.1 Enzymatic pathways involved in eicosanoid generation

#### 2.1.1 COX pathways

The COX pathway metabolizes free AA into bioactive prostanoids via sequential cyclooxygenase and peroxidase reactions (Smith et al., 2000; Vane et al., 1998; Patrignani and Patrono, 2015). The enzyme exists as two main isoforms: COX-1, encoded by a housekeeping gene (*PTGS1*), which maintains baseline prostanoid biosynthesis for tissue homeostasis, and COX-2, encoded by an immediate-early gene (*PTGS2*), induced by inflammatory or mitogenic stimuli. However, it is constitutively expressed in the CNS, vasculature, kidneys, and cancer cells (Patrignani and Patrono, 2015; Yamagata et al., 1993; Yasojima et al., 1999; Ricciotti and FitzGerald, 2011). Both isoforms oxidize free AA into the intermediate PGG_2_, which their intrinsic peroxidase activity rapidly reduces to PGH_2_ (Patrignani and Patrono, 2015). Downstream terminal synthases then convert PGH_2_ into specific prostanoids—including PGE_2_, PGD_2_, PGF_2α_, PGI_2_ (also known as prostacyclin), and TXA_2_—in a tissue-dependent manner (Patrignani and Patrono, 2015; Ricciotti and FitzGerald, 2011; Yagami et al., 2016). Among these, inducible microsomal PGE synthase-1 (mPGES-1) functionally couples with COX-2 in inflammatory and tumor microenvironments (TME) to serve as the predominant source of pathologically elevated PGE_2_ (Patrignani and Patrono, 2015; Ricciotti and FitzGerald, 2011). AA can bind to the cyclooxygenase site of COX-1 in at least three different orientations, leading mainly to the formation of PGG_2_ and, to a lesser extent, 11R-HpETE and 15R/S-HpETE that are reduced to the corresponding HETEs by glutathione peroxidases (Thuresson et al., 2000; Harman et al., 2004). Some AA products can be formed nonenzymatically, such as 5R-HETE, 8R- and 8S-HETE, 9R- and 9S-HETE, 11S-HETE, 12R-HETE, and 15R-HETE (Powell and Rokach, 2015; Mazaleuskaya et al., 2018).

Prostanoid responses are due to the activation of specific receptors, which belong to the G protein-coupled receptor (GPCR) family (Patrignani and Patrono, 2015; Ricciotti and FitzGerald, 2011; Narumiya and FitzGerald, 2001; Li et al., 2024). PGE_2_ signals through four receptor subtypes (EP1–EP4); EP4 is responsible for acute host defense, and EP2 and EP4 promote chronic inflammation, immune suppression, and tumor progression by activating the cAMP/PKA, PI3K/Akt, ERK/MAPK, and β-catenin pathways (Nakanishi and Rosenberg, 2013). EP4 serves as a dominant pro-tumorigenic driver via strong PI3K/Akt and ERK activation (Hawcroft et al., 2007), and EP3 functions through diverse splice variants (Narumiya and FitzGerald, 2001; Hawcroft et al., 2007). PGD_2_ signals through DP1 and DP2 receptors to exert divergent biological effects: vasodilation, platelet inhibition, anti-angiogenesis promoted by DP1, and allergic inflammation and pro-tumorigenic actions by DP2 (Narumiya and FitzGerald, 2001). PGF_2α_ acts through FP receptors to regulate smooth-muscle motility and luteolysis. At the same time, prostacyclin signals via IP, induces vasodilation, and opposes the pro-thrombotic and vasoconstrictive actions of TXA_2_ via TP receptors (Patrignani and Patrono, 2015; Ricciotti and FitzGerald, 2011).

The stroma, tumor-infiltrating leukocytes, or the cancer cells themselves can produce inflammatory mediators. Prominent among tumor-sustaining mediators is PGE_2_, a prostanoid lipid associated with enhanced cancer cell survival, growth, migration, invasion, angiogenesis, and immunosuppression (Wang and DuBois, 2010). COX-2 is often overexpressed in several human cancers, including colorectal, breast, stomach, lung, and pancreatic tumors (Dannenberg and Subbaramaiah, 2003). Several lines of evidence suggest that COX inhibitors reduce the risk of several cancers, including colorectal (Rothwell et al., 2011), gastric (Tian et al., 2010), breast cancer (Gierach et al., 2008), and melanoma (Gamba et al., 2013), supporting the role of COX-2 in cancer development. Because COX-2-dependent PGE_2_ is immunosuppressive, COX-2 inhibitors are useful additions to the arsenal of conventional and immune-based cancer therapies, most notably those based on immune checkpoint blockade.

#### 2.1.2 LOX pathways

LOXs are a family of non-heme iron proteins or dioxygenases, which catalyze the abstraction of hydrogen atoms from *bis*-allylic positions (1*Z*,4*Z*-pentadiene groups) of PUFA followed by stereospecific addition of dioxygen to generate hydroperoxides (Hajeyah et al., 2020). The human genome contains six functional LOX genes (*ALOX15*, *ALOX15B*, *ALOX12*, *ALOX12B*, *ALOX5*, and *ALOXE3*) expressed in various tissues (Snodgrass and Brüne, 2019; Ivanov et al., 2015). LOXs were traditionally named according to their positional specificity for AA (Kuhn et al., 2015). However, human 15-LOX-1 *(ALOX15*) exhibits dual reaction specificity with AA as a substrate, since 12S- and 15S-HpETE have been identified as oxygenation products (Kutzner et al., 2017). Thus, gene and enzyme names are recommended for hydroperoxides (Hajeyah et al., 2020; Kuhn et al., 2015). Cellular peroxidases can reduce HpETE to HETEs (Powell and Rokach, 2015). 5-LOX (*ALOX5*) is involved in LT biosynthesis from AA, a group of inflammation-derived lipid mediators that cause phagocyte chemotaxis and increased vascular permeability. First, 5-LOX catalyzes oxygenation of AA to 5S-HpETE, and further dehydration to the allylic epoxide LTA_4_. LTA_4_is then converted by LTA_4_ hydrolase to the dihydroxy acid LTB_4_, and by LTC_4_synthases to the glutathione conjugate LTC_4_. The other cysteinyl-LTs are formed by hydrolytic removal of γ-Glu and Gly from LTC_4_ (yielding LTD_4_and LTE_4_) (Haeggström, 2018). LTs induce cellular responses, such as smooth muscle contraction, phagocyte chemotaxis, and increased vascular permeability via GPCRs, BLT1/2 for LTB_4_and CysLT1/2 and GPR17 for the Cys-LTs (Yokomizo et al., 2018; Sasaki and Yokomizo, 2019).

Regulation of cellular LT biosynthesis involves intracellular migration of 5-LOX and cPLA_2_; in activated leukocytes, both enzymes associate with the nuclear membrane, where cPLA_2_releases AA from membrane phospholipids. The membrane-bound 5-LO-activating protein (FLAP) may facilitate the transfer of AA to 5-LOX. 5-LOX is expressed primarily in various leukocytes: polymorphonuclear leukocytes (neutrophils and eosinophils), monocytes/macrophages, mast cells, B-lymphocytes, dendritic cells, and foam cells of human atherosclerotic tissue. 5-LOX expression is frequently deregulated in solid and liquid tumors, and strong evidence shows that the enzyme plays an important role in carcinogenesis. However, recent data show that in addition to releasing oxylipins, 5-LOX can also influence gene expression in a lipid mediator-independent manner. 5-LOX produces 5-HETE, which is further converted by 5-HEDH to 5-oxo-HETE to regulate leukocyte activation and cell survival (Hajeyah et al., 2020; Grant et al., 2009). The human *ALOX12* gene encodes platelet-type 12-LOX (pl12-LOX; 12S-LOX) and is expressed in platelets, human pancreatic islets, keratinocytes, and certain tumor cell lines (Contursi et al., 2022). Pl12-LOX is the only LOX isoform present in platelets. Pl12-LOX oxygenates AA to produce 12S-HpETE, which is quickly converted to 12S-HETE by peroxidases. Upon platelet activation, the increase in intracellular calcium triggers the translocation of 12-LOX and the hydrolysis of PUFAs from the glycerolphospholipids via cPLA_2_ (Contursi et al., 2022). These events seem crucial for 12S-HETE generation in platelets. Approximately one-third of the 12-HETE generated by platelets is re-esterified into membrane phospholipids (PL-12-HETEs), forming compounds that enhance thrombin generation and clot formation and lead to procoagulant platelets (Contursi et al., 2021). The incorporation of HETEs into lipids has also been observed in many other cell types, including epithelial (Alpert and Walenga, 1993) and endothelial cells (Wang et al., 1990). 12-LOX is essential for FcγRIIa immune-mediated platelet activation. 12S-HETE activates the GPR31 receptor, promoting platelet functions, and may stimulate NADPH oxidases, increasing reactive oxygen species to activate further platelets (Guo Y. et al., 2011). Pl12-LOX and 12S-HETE are key regulators of ovarian cancer growth, with 12S-HETE supporting cell survival, invasion, motility, and angiogenesis (Guo A. M. et al., 2011). In prostate and skin cancers, 12S-HETE enhances cell adhesion to endothelial cells through αvβ3 integrins (Tang et al., 1993). Melanoma cells release 12S-HETE, causing reversible endothelial retraction via cytoskeletal changes (Contursi et al., 2022); it also promotes prostate cancer invasion by upregulating MMP9 through PI3K/Akt/NF-κB signaling and stimulating vascular endothelial growth factor (VEGF) secretion for angiogenesis. In colorectal cancer cells co-cultured with platelets, platelets transfer 12-LOX via EVs carrying active 12-LOX, allowing cancer cells to produce 12-HETE (Contursi et al., 2022). This molecule is primarily esterified in cancer cell phospholipids, promoting the expression of epithelial-mesenchymal transition (EMT) genes and supporting a pro-metastatic phenotype. Among the other LOXs, only 15-LOX-1 is briefly described here. Human 15-LOX-1, encoded by the *ALOX15* gene, primarily produces 15S-HETE and only minor amounts of 12S-HETE at an approximate 9:1 ratio (Kutzner et al., 2017). This enzyme plays complex and context-dependent roles, exhibiting both pro- and anti-inflammatory functions. Several studies have demonstrated the pro-inflammatory effects of 15-LOX-1 and its metabolites 12S-HETE and 15S-HETE (Cole et al., 2013; Li et al., 2013), while an anti-inflammatory role is often mediated through PPARγ activation (Sun et al., 2015; Thomas et al., 2025). Its functional outcome—beneficial or detrimental—depends on cell type, microenvironment, substrate availability, and interactions with other enzymes. Owing to these dual roles, ALOX15 has emerged as a therapeutic target in chronic inflammation, atherosclerosis, and cancer.

#### 2.1.3 CYP450 pathways

Traditionally linked to hepatic drug clearance and xenobiotic detoxification via oxidative elimination, CYP enzymes also metabolize AA through epoxygenase and hydroxylase pathways to generate potent autocrine and paracrine eicosanoid signaling molecules (Wang and Dubois, 2012; Bellien and Joannides, 2013; Tacconelli and Patrignani, 2014). The CYP epoxygenase pathway, driven mainly by CYP2C and CYP2J families, converts AA into four major regio-specific epoxyeicosatrienoic acids (5,6-, 8,9-, 11,12-, and 14,15-EET) (Wang and Dubois, 2012; Bellien and Joannides, 2013; Tacconelli and Patrignani, 2014). Primarily synthesized in the kidneys, vascular endothelium, heart, lungs, brain, and TME, EETs act as intracellular ligands for peroxisome proliferator-activated receptors (PPARs) to regulate lipid homeostasis (Mirza et al., 2019) while exerting vasorelaxant, anti-inflammatory, and angiogenic effects in the vasculature (Spector and Norris, 2007). CYP-derived EETs promote tumor proliferation, survival, invasion, metastasis, and angiogenesis, alongside modulating endothelial cell apoptosis, inflammation, progenitor cell migration, and capillary formation (Imig, 2018). Once formed, EETs are rapidly converted by soluble epoxide hydrolase (sEH) into dihydroxyeicosatrienoic acids (DHETs), which act as bioactive lipids that attenuate, diverge from, or directly oppose EET signaling depending on tissue context (Jiang et al., 2007). Conversely, the CYP4A and CYP4F ω-hydroxylase and ω-1-hydroxylase pathways generate 20-HETE alongside trace 16-, 17-, 18-, and 19-HETEs (Kroetz and Zeldin, 2002; Jiang et al., 2007). Unlike the largely protective EETs, 20-HETE is a potent pro-angiogenic and pro-proliferative mediator that acts primarily through its high-affinity GPCR, GPR75, to trigger EGFR autophosphorylation and downstream Raf/MEK/ERK and MAPK/NF-κB signaling cascades (Garcia et al., 2017; Jiang et al., 2007).

## 3 Eicosanoids in brain tumors

### 3.1 Physiological functions of eicosanoids in the CNS

Eicosanoids are key paracrine and autocrine signaling molecules in the CNS and have been implicated in numerous processes, including synaptic transmission, neurovascular coupling, sleep regulation, and tissue homeostasis (Jiang et al., 2007). Eicosanoid synthesis in the CNS is tightly regulated in a cell type-specific manner. In the healthy CNS, COX-1 is constitutively expressed throughout the brain and is predominantly associated with resident microglia. However, expression has also been reported in perivascular macrophages and, to a lesser extent, selected neuronal populations (Deininger and Schluesener, 1999; Yermakova et al., 1999). COX-2 is mainly expressed throughout the forebrain, with the highest levels in the hippocampus and cortex, and is confined to particular populations of excitatory neurons rather than glial cells (Yamagata et al., 1993; Kaufmann et al., 1996). By contrast, CYP enzymes are particularly abundant in astrocytes and cerebrovascular endothelial cells. The CYP pathway is particularly important for cerebrovascular regulation. Astrocytes abundantly express CYP epoxygenases that convert AA into EETs, potent vasodilatory mediators released following glutamate-induced intracellular Ca2+ elevations (MacVicar and Newman, 2015; Mishra et al., 2024). Conversely, CYP ω-hydroxylases generate 20-HETE, a potent vasoconstrictor produced mainly by vascular smooth muscle cells and pericytes (MacVicar and Newman, 2015; Mishra et al., 2024). The balance between EETs and 20-HETE is therefore a major determinant of neurovascular coupling and cerebral autoregulation. Components of the LOX pathway are expressed in neurons, microglia, astrocytes, and oligodendrocytes, although the physiological role of the LOX pathway in the healthy CNS remains poorly understood. Among the LOX isoforms, 5-LOX has been detected predominantly in neuronal populations of the hippocampus and cerebral cortex, as well as in subsets of microglia and astrocytes. In contrast, its activating protein FLAP appears to be strongly enriched in microglia (Lammers et al., 1996; Mi et al., 2020).

### 3.2 Functions of eicosanoids in brain tumors

Brain tumors remain one of the major unmet challenges in clinical oncology and, despite advances in diagnosis and treatment, the prognosis for affected patients remains poor. GBM, the most common and aggressive astrocytoma, is the most malignant and lethal primary brain tumor: even with standard therapy consisting of maximal surgical resection, radiotherapy, and chemotherapy, median survival is only about 15 months. Its invasive behavior, microenvironmental heterogeneity, and resistance to therapy make a deeper understanding of its biology essential for developing new therapeutic strategies, and eicosanoids have emerged as important players in this context. In GBM, the orderly compartmentalization of AA metabolism seen in the healthy brain breaks down. Tumor cells, glioma stem-like cells, endothelial cells, reactive astrocytes, microglia, infiltrating macrophages, and circulating blood components all contribute to a shared eicosanoid milieu. Through the COX, LOX, and CYP450 pathways, AA-derived mediators regulate several hallmarks of glioma biology, including proliferation, survival, invasion, stemness, angiogenesis, immune escape, and resistance to therapy. The evidence, however, is uneven across pathways: the COX-2–PGE_2_ axis is comparatively well characterized, whereas the biological significance of LTs, HETEs and EETs is still less clearly defined. The following sections therefore focus on the enzymatic pathways most extensively studied to date.

### 3.3 The COX pathway in brain tumors

COX-2 is broadly overexpressed in brain tumors. Elevated COX-2 expression has been reported in astrocytomas, GBMs, meningiomas, medulloblastomas, craniopharyngiomas, oligodendrogliomas, ependymomas and neurinomas, and most human malignant glioma cell lines constitutively express high levels of the enzyme (Qiu et al., 2017). Consistent with the tumor as a major source of prostanoids, surgical resection reduces plasma PGE_2_ concentrations in patients with malignant, but not benign, brain tumors (Loh et al., 2002); moreover, the downstream PG machinery is also upregulated in gliomas. PGES isoforms are overexpressed in gliomas and increase with tumor grade. Recurrent tumors express more COX-2, mPGES-1, and cPGES than primary tumors (Mattila et al., 2009). Collectively, these findings demonstrate that activation of the COX-2/mPGES-1/PGE_2_ axis is a characteristic feature of malignant glioma. Increased COX-2 expression correlates with higher tumor grade, enhanced invasiveness, earlier radiological recurrence, and reduced overall survival in patients with GBM (Qiu et al., 2017; Shono et al., 2001; Sminia et al., 2005; Buccoliero et al., 2006; Onguru et al., 2008; Jiang et al., 2017; Tsai et al., 2022). In one study, patients with COX-2-negative GBMs survived more than twice as long as those with COX-2-positive tumors, and all patients surviving longer than 24 months had COX-2-negative lesions (Buccoliero et al., 2006). Furthermore, elevated COX-2 expression together with increased PGE_2_ production has repeatedly been associated with poor clinical outcome (Lombardi et al., 2022; Dean and Hooks, 2023), supporting COX-2 as an independent prognostic marker of glioma aggressiveness. Among prostanoids, PGE_2_ is considered the principal downstream effector of COX-2 in GBM. Acting primarily through the EP2 and EP4 receptors, PGE_2_ activates multiple intracellular signaling pathways, including PI3K/Akt, MAPK/ERK, and Wnt/β-catenin, thereby promoting cell-cycle progression, proliferation, survival, and resistance to apoptosis (Lombardi et al., 2022; Dean and Hooks, 2023; Wu et al., 2022). PGE_2_ also activates growth-factor pathways. In irradiated glioma cells, it transactivates EGFR, promoting tumor-cell survival and proliferation (Brocard et al., 2015). COX-2/PGE_2_–EGFR crosstalk likely contributes to GBM progression *in vivo*. COX-2-derived PGE_2_ also maintains glioma stem-like cells (GSCs) by inducing inhibitor of DNA binding 1 (Id1), a marker of highly tumorigenic, self-renewing, therapy-resistant GSCs. Activation of EP2 and EP4 receptors increases Id1 expression, tumor sphere formation, and self-renewal capacity, whereas pharmacological or genetic inhibition of the pathway suppresses these stem-cell phenotypes (Cook et al., 2016). This mechanism appears particularly important following radiotherapy, as irradiation stimulates PGE_2_ production, promoting survival and expansion of Id1-positive cells. Consequently, radiotherapy may inadvertently trigger a lipid-mediated adaptive response that facilitates repopulation of the residual tumor. GBMs display abnormal vasculature, vascular leakage, and hypoxia that promote tumor progression. COX-2 expression correlates with VEGF and microvessel density, while PGE_2_ increases VEGF production, indicating that COX-2-driven angiogenesis is largely mediated by PGE_2_ (Xu and Shu, 2007). In addition, EP2/EP4 signaling promotes angiogenesis beyond VEGF induction, and dual blockade is more effective than inhibiting either receptor alone (Dean and Hooks, 2023; Finetti et al., 2020; Qiu et al., 2023). Immune evasion represents another hallmark of COX-2/PGE_2_ signaling in GBM. The hypoxic TME induces COX-2 expression (Tafani et al., 2011), while chemotherapy and radiotherapy further stimulate PGE_2_ synthesis, promoting immunosuppressive cytokines such as IL-6, IL-10, and GM-CSF and limiting T-cell infiltration and proliferation. EP2/EP4 signaling promotes angiogenesis beyond VEGF induction, and dual blockade is more effective than inhibiting either receptor alone (Finetti et al., 2020; Holt et al., 2011). In parallel, PGE_2_ promotes polarisation of tumor-associated macrophages (TAMs) and microglia toward an immunosuppressive M2-like phenotype, reinforcing the tumor-supportive immune microenvironment (Finetti et al., 2020; Shen et al., 2026). This interaction is bidirectional, as glioma-derived soluble factors induce COX-2 and mPGES-1 expression in microglia, establishing a paracrine feedback loop that further amplifies PGE_2_ production within the TME (Nakano et al., 2008). Beyond its effects on proliferation and immune regulation, the COX-2/PGE_2_ axis contributes substantially to GBM invasion. Activation of EP receptors stimulates PI3K/Akt and MAPK/ERK signaling, increasing tumor-cell migration and invasion while inducing matrix metalloproteinases (MMPs) that degrade the extracellular matrix (Dean and Hooks, 2023; Ferreira et al., 2020). Upstream signaling pathways converge on this axis: reactive oxygen species activate ERK signaling to induce COX-2/PGE_2_-dependent migration in U87 cells (Chiu et al., 2010), whereas Nuclear factor of activated T-cells, cytoplasmic 1 (NFATC1) enhances the invasive phenotype of U251 glioma cells by transcriptionally upregulating COX-2 (Lu-Emerson et al., 2015). Finally, increasing evidence implicates the COX-2/PGE_2_ pathway in both intrinsic and acquired resistance to therapy. Temozolomide (TMZ), the standard chemotherapeutic agent for GBM (Stupp et al., 2005; Strobel et al., 2019), is frequently limited by acquired resistance (Oliver et al., 2020). Resistant glioma models display extensive remodeling of AA metabolism, suggesting that alterations in lipid metabolism contribute to treatment failure (Kao et al., 2023). In this context, PGE_2_ promotes mitochondrial fatty-acid oxidation, tricarboxylic acid cycle activity, and survival signaling through EP1 and EP3 receptors, whereas inhibition of these receptors sensitizes tumor cells to TMZ (Tsai et al., 2022). The strongest evidence concerns radiation-induced PGE_2_ signaling. Following irradiation, glioma cells release PGE_2_, which activates EGFR signaling and enhances Id1-dependent self-renewal, promoting survival and repopulation of residual tumor cells (Brocard et al., 2015). Collectively, experimental and clinical evidence identifies the COX-2/mPGES-1/PGE_2_ axis as a central driver of GBM progression. By regulating tumor proliferation, stemness, angiogenesis, immune evasion, invasion, and therapeutic resistance, this pathway contributes to multiple hallmarks of malignancy and represents an attractive therapeutic target.

#### 3.3.1 Clinical evidence of the efficacy of NSAIDs and aspirin in brain tumor

Long-term use of nonsteroidal anti-inflammatory drugs (NSAIDs), particularly aspirin and selective COX-2 inhibitors (coxibs), has been extensively studied for cancer chemoprevention, especially in colorectal cancer (CRC); epidemiological and experimental studies suggest reduced risk of colonic tumor development (Patrignani and Patrono, 2015; Patrigna et al., 2016; Patrono, 2023). NSAIDs are among the most widely used therapeutic agents worldwide and comprise a chemically diverse group of compounds (Patrignani and Patrono, 2015) that inhibit prostanoid synthesis by targeting COXs (FitzGerald and Patrono, 2001; Simmons et al., 2004). They are classified by COX-1/COX-2 selectivity, typically assessed using *in vitro* and *ex vivo* human whole-blood assays that measure inhibitory effects on platelet COX-1 and monocyte COX-2 activity (Patrignani et al., 1994; Patrono et al., 1980). Traditional NSAIDs inhibit both COX-1 and COX-2 at therapeutic doses, whereas coxibs selectively inhibit COX-2. Unlike traditional NSAIDs and coxibs, aspirin displays a unique pharmacodynamic trait: it irreversibly inhibits COX-1 and COX-2 by transferring its acetyl group to serine 529 and 516, respectively, thus preventing AA access to the catalytically active site of the COX channel. This effect results in persistent inhibition of platelet COX-1-dependent TXA_2_ biosynthesis throughout the dosing interval (24 h), despite the drug’s short half-life, because platelets have limited capacity for *de novo* protein synthesis as anucleated cells (Patrono et al., 2001). Low-dose aspirin (75–100 mg/day) preferentially affects platelet COX-1, resulting in complete inhibition of platelet TXA_2_ biosynthesis with limited effects on extraplatelet COX activity (Patrignani et al., 2014; Patrignani et al., 1982). Traditional NSAIDs and coxibs are effective in CRC chemoprevention (Wang and DuBois, 2010; Cha et al., 2007; Garcia Rodriguez et al., 2013). A most plausible mechanism involves their shared capacity to inhibit COX-2-dependent PGE_2_, which has a predominant role in promoting CRC growth (Wang and DuBois, 2010); PGE_2_ modulates several signal transduction pathways that affect proliferation, programmed cell death (apoptosis), angiogenesis, immune response, cellular adhesion, differentiation, and tumor invasion (Wang and DuBois, 2010). Regarding aspirin, randomized clinical trials (RCTs) have recently been conducted in patients with resected early-stage CRC to evaluate the effects of aspirin versus placebo on disease-free survival and overall survival (Martling et al., 2025; Patrignani et al., 2026; Patrono et al., 2026). In the ALASCCA trial, aspirin (160 mg/day) reduced CRC recurrence by 51% in patients with *PIK3CA* hotspot mutations in exons 9 or 20 (primary endpoint) and by 58% in those with other somatic alterations in the PI3K/PTEN pathway. A recent meta-analysis of three RCTs (SAKK 41/13, ALASCCA, and ASCOLT-TR) comparing adjuvant aspirin with placebo in CRC patients with *PIK3CA* mutations demonstrated a significant reduction in DFS events (Segelov et al., 2026).

The clinical and epidemiological evidence linking NSAIDs to reduced cancer risk has historically been robust for gastrointestinal cancer, but deeply inconsistent regarding neuro-oncological conditions such as glioma (Amirian et al., 2019). To date, only one randomized controlled trial has evaluated the effects of low-dose aspirin on brain tumors (Cook et al., 2005); the study enrolled 39,876 US women aged ≥45 years without prior cancer and randomized them to aspirin (100 mg every other day) or placebo, with a mean follow-up of 10.1 years. During follow-up, 31 brain tumors were reported, including 17 in the aspirin group [Relative risk (RR): 1.20 (95% CI: 0.60–2.46)], suggesting that low-dose aspirin was not associated with a significant reduction in brain tumor risk (Cook et al., 2005). A landmark investigation by the Glioma International Case-Control Study (GICC) consortium, analyzing a large number of cases and controls (4,533 cases and 4,171 controls), demonstrated that regular daily aspirin consumption for 6 months or longer was associated with a significant 38% reduction in glioma risk Odds Ratio [OR]: 0.62; 95% CI: 0.54–0.70) (Amirian et al., 2019). This association showed a significant duration-response relationship (P = 1.67 × 10^−17^), suggesting that longer regular aspirin use was associated with stronger protective effects. Conversely, no protective trends or duration-dependent benefits were observed for other NSAIDs (Amirian et al., 2019). When combined with previous case-control studies, a marginally significant association was found between aspirin use and glioma risk (OR: 0.84; 95% CI: 0.70–1.02) (Amirian et al., 2019).

Clinical evaluation of COX-2 inhibition in brain tumors remains limited. The Phase II trial NCT00068770 investigated celecoxib in patients with newly diagnosed GBM receiving radiotherapy and anticonvulsants. Still, it was terminated early following the establishment of the Stupp protocol, which introduced TMZ as standard of care and rendered the original design obsolete. Other Phase II trials (NCT00047281 and NCT00047294) evaluated whether adding celecoxib could enhance the efficacy of aggressive brain tumor regimens; however, their results were not published due to limited therapeutic benefit and substantial treatment-related toxicity, including thalidomide-associated deep vein thrombosis and neuropathy (Chen et al., 2013; Chen H. C. et al., 2023). One possible explanation for the limited efficacy of oral celecoxib is insufficient intratumoral drug exposure. Although celecoxib is lipophilic and can cross the BBB, the concentrations achieved in the tumor following oral administration may have been insufficient to exert a meaningful therapeutic effect.

#### 3.3.2 COX-2/mPGES-1/PGE_2_ signaling and emerging therapeutic strategies in cancer and glioma

Although the epidemiological evidence supporting aspirin and other NSAIDs for glioma prevention remains inconsistent, a large body of experimental evidence indicates that dysregulation of the COX-2/PGE_2_ signaling axis is a key driver of tumor progression and represents an attractive therapeutic target. In GBM, elevated expression of both COX-2 and mPGES-1 has been associated with higher tumor grade, increased invasiveness, enhanced vascularization, and poor patient prognosis, highlighting the importance of this pathway within the immunosuppressive TME.

Although selective COX-2 inhibitors demonstrated significant antitumor activity in preclinical models and early clinical studies, their long-term clinical use has been limited by an increased risk of cardiovascular events, largely attributable to suppression of endothelial PGI_2_ biosynthesis while sparing platelet TXA_2_ production (FitzGerald, 2004; Grosser et al., 2005). This limitation has stimulated the development of alternative strategies targeting downstream components of the pathway. Among these, mPGES-1 has emerged as an attractive therapeutic target because its inhibition selectively reduces pathological PGE_2_ production while largely preserving the synthesis of other prostanoids, including the vasculoprotective prostacyclin, which can also be increased. Genetic deletion or pharmacological inhibition of mPGES-1 suppresses tumor growth, angiogenesis, metastasis, cancer stem cell maintenance, and inflammatory signaling in multiple experimental cancer models while exhibiting a more favorable cardiovascular safety profile than COX-2 inhibition (Larsson and Jakobsson, 2015). Advances in structural biology and structure-guided drug design have accelerated the development of selective human mPGES-1 inhibitors with improved potency and pharmacokinetic profiles. Several chemical classes, including indole derivatives, benzoxazoles, phenanthrene imidazoles, pirinixic acid analogs, and dual COX-2/mPGES-1 inhibitors, suppress PGE_2_ synthesis and inhibit tumor cell proliferation, angiogenesis, and metastasis in preclinical models (Koeberle and Werz, 2015; Xu et al., 2008). Nevertheless, clinical translation of mPGES-1 inhibitors has progressed slowly because structural differences between human and rodent mPGES-1 complicate preclinical evaluation, and because optimizing drug exposure, target engagement, and clinical efficacy remains challenging. In neuro-oncology, effective BBB penetration is another key consideration for next-generation compounds. An alternative approach is to block PGE_2_ signaling through selective EP receptor antagonism. EP4 antagonists have attracted particular interest because EP4 is a key mediator of PGE_2_-driven immunosuppression within the TME. Preclinical studies show that agents such as grapiprant, E7046, and the dual EP2/EP4 antagonist TPST-1495 enhance dendritic cell activation, increase CD8^+^ T-cell infiltration, reduce immunosuppressive myeloid cell accumulation, and improve responses to immune checkpoint blockade (Francica et al., 2023). TPST-1495, the most clinically advanced dual EP2/EP4 antagonist, has shown acceptable safety and early pharmacodynamic evidence of immune activation, supporting further evaluation in combination with PD-1/PD-L1 inhibitors, chemotherapy, and targeted therapies. Collectively, these findings suggest that mPGES-1 inhibition or EP2/EP4 blockade may overcome PGE_2_-mediated immunosuppression while avoiding some cardiovascular limitations of COX-2 inhibition. Although clinical evidence remains limited, these approaches hold promise, particularly for GBM, where overcoming the immunosuppressive TME remains a major therapeutic challenge.

### 3.4 The LOX pathway in brain tumor

The 5-LOX pathway represents the second most extensively studied pathway of AA metabolism in GBM. ALOX5 (5-LOX) expression is significantly elevated in GBM compared with non-neoplastic brain tissue (Golubic et al., 2003; Nathoo et al., 2006). Within the TME, 5-LOX is expressed not only by glioma cells but also by TAMs and microglia (Golubic et al., 2003; Nathoo et al., 2006), with particularly high expression observed in GSCs, where it contributes to the maintenance of stemness and self-renewal (Wang et al., 2011). Pharmacological inhibition of 5-LOX reduces tumor-sphere formation, alters cellular metabolism, and suppresses the tumorigenic potential of GSCs, highlighting its importance in sustaining the therapy-resistant stem-cell compartment (Wang et al., 2011). 5-LOX activity and its principal metabolite, 5-HETE, are elevated in gliomas, and enhanced expression of other components of the LT biosynthetic pathway, including FLAP (gene name: *ALOX5AP)* and LTA_4_ hydrolase (gene name: *LTA4H*), has been reported in high-grade gliomas (Ko et al., 2023). Increased expression of these enzymes correlates with tumor grade, peritumoral edema, and poor clinical outcome. At the same time, co-expression of COX-2 and 5-LOX has been proposed as an independent prognostic marker of adverse survival in GBM (Wang et al., 2015). Beyond tumor progression, LT signaling may also contribute to gliomagenesis, with evidence suggesting roles in the earliest stages of malignant transformation. Supporting this possibility, the “GA” (heterozygous) genotype of the rs2291427 single-nucleotide polymorphism (SNP) in the *ALOX5* gene has been significantly associated with an increased risk of GBM (Jin et al., 2013). Functionally, the 5-LOX pathway regulates several hallmarks of GBM malignancy. In the tumor immune microenvironment, 5-HETE released by ALOX5-overexpressing glioma cells promotes recruitment of tumor-associated macrophages, enhances PD-L1 expression, and drives macrophage polarisation toward an immunosuppressive M2-like phenotype, thereby facilitating immune evasion (Chen et al., 2024). Accordingly, transcriptomic analyses associate *ALOX5AP* expression with macrophage-rich, immunosuppressive glioma subtypes and poor clinical outcomes but cannot distinguish LT production by tumor versus infiltrating myeloid cells (Song et al., 2024). In addition to its immunomodulatory functions, 5-LOX signaling contributes to GSC maintenance and promotes tumor proliferation and survival (Wang et al., 2011). Besides 5-LOX, the 15-LOX/*ALOX15* pathway has also been implicated in GBM biology. Increased *ALOX15* expression and 15S-HETE production have been associated with cytoskeletal remodeling, tumor-cell motility, and angiogenesis (Zhang et al., 2005; Lim et al., 2019; Souza et al., 2020).

#### 3.4.1 LOX inhibitors in brain cancer

The aberrant overexpression of 5-LOX in GBM, but not in normal brain tissue, highlights the 5-LOX pathway as a promising therapeutic vulnerability in high-grade gliomas (Nathoo et al., 2006; Ishii et al., 2009; Tang et al., 2023). Depletion of *Alox5* in mice impairs GBM cell growth and enhances sensitivity to TMZ by downregulating β-catenin levels. Wnt agonist treatment reverses this growth inhibition, confirming a Wnt/β-catenin-dependent mechanism (Tang et al., 2023). Across diverse cancers, 5-LOX-derived lipid mediators, including 5-HETE, 5-oxo-eicosatetraenoic acid (5-oxo-ETE), and Cys-LTs, regulate tumor cell survival, proliferation, migration, inflammatory signaling, and metastasis. However, while 5-LOX-derived 5-HETE promotes gastric carcinoma cell proliferation via mitogen-activated protein kinase (MEK/ERK) activation independently of Akt signalling, in high-grade glioma it primarily regulates the tumor immune landscape and extracellular matrix within the TME (Tang et al., 2021); glioma-derived 5-HETE correlates with increased Programmed Death-Ligand 1 (PD-L1) expression, M2-like macrophage polarization, and enhanced recruitment and migration of glioma-associated microglia (GAMs), key mediators of tissue remodelling and immune evasion (Chen et al., 2024). Concurrently, downstream LT signaling via CysLT1 actively facilitates glioma cell migration and parenchymal invasion by driving the enzymatic expression of MMP-2 and MMP-9 (Piromkraipak et al., 2018). Despite these insights, the comprehensive mechanistic networks governed by 5-LOX overexpression in glioma pathogenesis remain only partially characterized. Thus, the targeted pharmacological inhibition of 5-LOX has emerged as a promising therapeutic strategy for GBM. Preclinical investigations using the small-molecule 5-LOX inhibitor (approved for asthma), zileuton, have demonstrated considerable translational potential, particularly in combination regimens; co-administration of zileuton and TMZ exerts a distinct synergistic therapeutic effect in murine models (Tang et al., 2023). This chemosensitization is mediated primarily via the downstream downregulation of β-catenin signaling, thereby dampening canonical Wnt-driven survival programs. Beyond direct antineoplastic efficacy, 5-LOX inhibition exhibits broader CNS immunomodulatory effects; in experimental mouse models of traumatic brain injury, zileuton administration mitigated profound neuroinflammation by arresting LT release and suppressing pro-inflammatory cytokine production (Xu et al., 2025). Collectively, these preclinical milestones highlight zileuton’s dual capacity to enhance chemotherapeutic responsiveness and modulate deleterious CNS inflammatory dynamics, strongly warranting further rigorous clinical validation of 5-LOX inhibition strategies in GBM management.

Given the complex biological network of the 5-LOX cascade, evaluating downstream receptor targets is critical for comprehensive therapeutic intervention; elevated CysLT1 levels have been documented in high-grade gliomas (Vivithanaporn et al., 2020). This clinical observation suggested investigating the LT receptor antagonists, montelukast and zafirlukast, in GBM therapy. *In vitro* evaluations by Piromkraipak et al. demonstrated that both montelukast and zafirlukast successfully restricted the migratory and invasive capacities of A172 and U373 glioma cell lines (Piromkraipak et al., 2018). This anti-invasive effect was mediated by CysLT1 inhibition, with reduced MMP-2 and MMP-9 expression. LT receptor antagonists also decreased Bcl-2 expression and ERK1/2 phosphorylation without affecting Bax, implicating the CysLT1–ERK/Bcl-2 axis in their anti-tumorigenic activity. Beyond receptor antagonism, multi-target compounds with potent 5-LOX inhibitory activity, including the dual 5-LOX/mPGES-1 inhibitor embelin, have also been investigated; embelin is a naturally occurring para-benzoquinone derivative recognized as a dual inhibitor of both 5-LOX and mPGES-1 (Schaible et al., 2013). The embelin derivative RF-ID has demonstrated promising cytotoxic efficacy, inducing apoptosis in approximately 30% of treated U87MG and LN229 GBM cells. However, because RF-ID concurrently downregulates key genes within the Inhibitor of Apoptosis Protein (IAP) and NFkB families, the precise proportional contribution of 5-LOX inhibition to its overall therapeutic efficacy remains to be fully elucidated. In addition, Morin et al. (2017) have found that caffeic acid phenethyl ester (CAPE) and curcumin possess strong, though distinct, 5-LOX inhibitory profiles. In HEK293 cell models, treatment with 1 µM of CAPE suppressed 5-LOX activity by 75%, whereas curcumin exhibited a dose-dependent mitigation of the enzyme, yielding a 65% reduction at a concentration of 5 μg/mL. However, this did not linearly translate into a reduction of glioma cells in Hs683 and LN319 cell lines, as CAPE derivatives showed varying reductions in glioma cell viability, suggesting CAPE’s cytotoxic actions may be independent of its inhibition of 5-LOX. Concurrently, while curcumin achieved a 68% reduction in cell survival across both GBM models, it remains unclear whether these off-target effects are driven uniquely by 5-LOX cascade disruption or result from broader, non-specific multi-kinase inhibition. Consequently, further research is needed to determine whether CAPE and curcumin are feasible anti-cancer compounds in a GBM context, and to what extent their therapeutic potential is mediated by 5-LOX inhibition (Morin et al., 2017).

The introductory step in LT biosynthesis involves the oxidation of AA to 5-HpETE, a process critically dependent upon the functional coupling of 5-LOX with FLAP at the nuclear envelope. Given the established role of downstream LT signaling in sustaining tumor cell survival and mitigating apoptotic pathways, the targeted disruption of the 5-LOX/FLAP complex presents a potential target. The FLAP antagonist MK886 has antineoplastic efficacy in non-central nervous system malignancies, including gastric carcinoma (Fan et al., 2004). In addition, MK886 exerts a robust, dose-dependent restriction on glioma cell proliferation (Lim et al., 2010) and triggers apoptotic cascades in 5-LOX-expressing U-87MG and A172 cell lines; conversely, 5-LOX-deficient U373 cells exhibited no significant reduction in tumor cell viability upon treatment, strongly indicating that its cytotoxic properties are fundamentally mediated via the disruption of the 5-LOX pathway. Intriguingly, this phenotypic response occurred without parallel alterations in baseline FLAP expression levels, suggesting that the precise intracellular pharmacology of MK886 may operate through mechanisms independent of direct FLAP protein downregulation (Lim et al., 2010). While some evidence suggests FLAP inhibitors have therapeutic potential in other cancers (Sarveswaran et al., 2010; Cianchi et al., 2006), we do not know this in a GBM context. This underexplored area merits investigation in GBM models, as FLAP’s role in MK886s mechanism remains unclear. 12-HETE promotes migration in U87 and U251 GBM cells; ML355, a 12-LOX inhibitor, reduced 12-HETE production and reversed the pro-migratory phenotype (Yang et al., 2021). However, to our knowledge, ML355 has not been studied as a direct therapeutic agent in GBM management.

Currently, the flavone baicalein is the most widely documented 12-LOX inhibitor directly evaluated in glioma. The comparative efficacy of baicalein and the nonselective lipoxygenase inhibitor nordihydroguaiaretic acid (NDGA) was studied in rat C6 glioma cells (Terpinskaya et al., 2021); baicalein did not show significant cytotoxic or antiproliferative effects within the initial 24 h of administration, yet exerted a robust restriction on cell proliferation after a prolonged 72-h exposure. Conversely, NDGA reduced cancer cell viability by 18% within the first 24 h. However, baicalein can inhibit not only 12-LOX but also 15-LOX and CYP2C9 (Si et al., 2009); thus, assumptions about therapeutic targeting of 12-LOX in glioma cannot be based on evidence from baicalein alone. However, to our knowledge, no specific 12-LOX inhibitor has been tested in GBM.

Pharmacological inhibition of 15-LOX (using luteolin) impairs GBM cell migration, proliferation, and metabolism. However, effects vary across models, reflecting eicosanoid signaling influenced by tumor subtype, receptor expression, and the balance between pro-tumorigenic and pro-resolving pathways (Souza et al., 2020).

#### 3.4.2 Reduced resistance to chemotherapy by inhibiting COX-2 and 5-LOX

Chemoresistance represents one of the major challenges in cancer therapy and is increasingly recognized as a consequence of the interplay between tumor cell-intrinsic mechanisms and the inflammatory TME. Among the inflammatory pathways involved, COX-2 and 5-LOX, the key enzymes of AA metabolism, have emerged as central regulators of resistance to chemotherapy. Aberrant COX-2 expression across solid tumors elevates PGE_2_ to hyperactivate PI3K/Akt, MAPK/ERK, NF-κB, and STAT3 pathways, driving cell proliferation, anti-apoptotic signaling, EMT, cancer stem cell maintenance, and metastasis (Lin et al., 2019; Hidalgo-Estévez et al., 2020; Li et al., 2020). Notably, COX-2 upregulation also occurs as an adaptive response to chemotherapy-induced DNA damage, where increased PGE_2_ reshapes the TME into an immunosuppressive state by impairing dendritic cell activation and CD8^+^ T-cell responses while accumulating myeloid-derived suppressor cells (MDSCs), regulatory T cells, and M2 tumor-associated macrophages (Li et al., 2020; Bell et al., 2022). Mechanistically, COX-2-derived PGE_2_ acts via EP2 and EP4 receptors to upregulate anti-apoptotic proteins (Bcl-2, Bcl-xL, survivin), suppress caspases, and induce ATP-binding cassette (ABC) transporters (ABCB1, ABCG2) to enhance drug efflux (Li et al., 2020; Bell et al., 2022). Selective COX-2 inhibition with celecoxib restores sensitivity to platinum compounds, taxanes, fluoropyrimidines, and anthracyclines while enhancing chemo-immunotherapy responses (Lin et al., 2019; Li et al., 2020; Bell et al., 2022). Concurrently, the 5-LOX pathway contributes to resistance through LTB_4_/BLT signaling, which activates PI3K/Akt, ERK1/2, and NF-κB to promote DNA repair, oxidative stress adaptation, cytokine release, and apoptosis resistance (Meshram et al., 2021). Because selective COX-2 inhibition can trigger a compensatory metabolic shunt toward 5-LOX and LT biosynthesis, dual COX-2/5-LOX inhibition has emerged as a superior strategy to block PG and LT production simultaneously, suppress downstream NF-κB/STAT3/Akt networks, reduce cancer stemness and multidrug resistance transporters, and overcome inflammation-mediated chemoresistance (Li et al., 2020; Meshram et al., 2021).

### 3.5 The CYP pathway in brain tumor

The CYP450 pathway is less studied in GBM, and the available evidence points mainly to a pro-tumoral role for 20-HETE. Direct data on 20-HETE production in GBM are scarce; the mediator appears to be generated chiefly by TAMs and endothelial progenitor cells (Wang et al., 2017a) and has been shown to increase GBM cell proliferation (Guo et al., 2008). Functional support comes from overexpression studies: high CYP4A1 expression in U251 glioma cells markedly increased 20-HETE production, enhanced proliferation *in vitro*, and generated larger intracranial tumors in rats, indicating a direct tumor-promoting role for the CYP4A–20-HETE pathway. Because CYP4A1 is a rodent enzyme, however, the corresponding human CYP4F enzymes require more careful study in patient-derived GBM models (Guo et al., 2008). Beyond proliferation, both EETs and 20-HETE signaling have been linked to tumor angiogenesis, although specific evidence from human GBM remains limited (Sorokin and Alexanian, 2013).

Emerging evidence demonstrates that CYP-derived EETs regulate critical oncogenic processes, driving primary tumor growth, progression, and metastasis by promoting cell proliferation, survival, invasion, and angiogenesis (Wang B. et al., 2021). CYP epoxygenases such as CYP2J2 are highly expressed across various human carcinoma cell lines—including LS-174 (colon adenocarcinoma), ScaBER (bladder squamous cell carcinoma), SiHa (cervical squamous cell carcinoma), U251 (GBM astrocytoma), A549 (lung alveolar basal epithelial carcinoma), Tca-8113 (tongue squamous cell carcinoma), NCL-H446 (small cell lung carcinoma), and HepG2 (hepatocellular carcinoma). Beyond the CYP2J subfamily, members of the CYP2C family, predominantly CYP2C8 and CYP2C9, are functionally expressed in a variety of human cancer models, including breast (MCF-7), liver (HepG2), and lung (A549) carcinomas, and human GBM multiforme models (U251 and U87MG), where they actively contribute to endogenous EET production (Wang B. et al., 2021). *In vitro*, CYP epoxygenase overexpression or exogenous treatment with 11,12- and 14,15-EET markedly enhances the migration, invasion, and pro-metastatic gene expression profiles of diverse cell lines (specifically Tca-8113, A549, HepG2, and LS-174), a phenotype fundamentally driven by the upregulation of CD44 and MMPs (Jiang et al., 2007). Supporting these findings *in vivo*, CYP2J2 overexpression in xenografted MDA-MB-231 cells significantly accelerates pulmonary dissemination, resulting in a ∼60% increase in lung metastatic nodules in athymic BALB/c mice (Jiang et al., 2007). Similarly, upregulation of the CYP2C pathway yields comparable oncogenic effects. Human carcinoma models expressing CYP2C8 and CYP2C9 exhibit enhanced proliferation, migration, and invasiveness driven by the intracellular accumulation of 11,12- and 14,15-EET, which trigger EGFR transactivation and downstream PI3K/Akt pro-survival signaling (Jiang et al., 2007). Beyond the pro-angiogenic activity of CYP epoxygenase-derived EETs, AA undergoes regiospecific hydroxylation catalyzed predominantly by members of the CYP4A and CYP4F subfamilies, most notably CYP4A11, CYP4F2, and CYP4F3 in humans (Wang B. et al., 2021). This pathway leads to the formation of HETEs, mainly 20-HETE (Pascale, 2023). As a potent lipid mediator, 20-HETE plays a pivotal role in regulating vascular tone, cellular mitogenesis, and TME remodeling (Elshenawy et al., 2017). At the cellular level, 20-HETE functions as a key mitogenic driver. Early *in vitro* experiments established that 20-HETE induces rapid cell proliferation, DNA synthesis, and cell cycle progression in vascular smooth muscle cells and renal proximal tubule cultures (Zhang C. et al., 2018). These mitogenic responses are largely transduced via activation of GPCRs, specifically GPR75 (Cárdenas et al., 2020), which trigger downstream signaling cascades including Protein Kinase C activation, NADPH oxidase-mediated reactive oxygen species (ROS) generation, transactivation of EGFR, and downstream signaling through the Ras/Raf/MEK/ERK and PI3K/Akt pathways, preventing apoptosis under hypoxic tumor conditions (Zhang C. et al., 2018). In the TME, 20-HETE is a primary driver of pathological angiogenesis, directly stimulating endothelial cell proliferation, migration, and capillary tube formation (Sorokin and Alexanian, 2013). Its pro-angiogenic effects are driven by VEGF upregulation. In endothelial and stromal cells, 20-HETE induces VEGF expression and secretion, creating autocrine/paracrine loops that increase microvessel density, vascular permeability, and tumor growth (Sorokin and Alexanian, 2013). Dysregulation and overexpression of CYP4A and CYP4F enzymes have been documented across various malignancies, including human renal, breast, brain, and gastrointestinal cancers, where elevated 20-HETE levels correlate with higher tumor grade, increased invasiveness, and poor prognosis (Sorokin and Alexanian, 2013). Consequently, targeting the CYP4A/F–20-HETE pathway represents a compelling therapeutic strategy. Indeed, small-molecule inhibitors of 20-HETE synthesis, such as HET0016, and GPR75 antagonists have demonstrated significant preclinical efficacy—suppressing tumor vascularization, reducing primary tumor growth, and blunting metastatic dissemination (Pascale, 2023; Cárdenas et al., 2023). For instance, HET0016, a selective inhibitor of CYP4A, effectively blocks EGFR phosphorylation and reduces U251 glioma cell proliferation, directly linking the CYP4A/20-HETE signaling axis to glioma progression in preclinical models. Further supporting this mechanism, ectopic transfection of these cells with CYP4A1 cDNA results in a two-fold increase in cell proliferation, along with increased 20-HETE biosynthesis (Guo et al., 2008). While the exact role of the EET arm in GBM is less well-characterized, targeting parallel eicosanoid pathways shows great promise; a dual inhibitor of sEH and COX-2 (named PTUPB) successfully suppressed GBM growth and tumor angiogenesis by reducing EGFR expression (Li et al., 2017).

### 3.6 Translational perspectives for eicosanoid-targeted therapies

Overall, GBM heterogeneity makes it unlikely that targeting a single eicosanoid pathway will be sufficient to achieve substantial therapeutic benefit. Rather than replacing current treatments, pharmacological modulation of eicosanoid signaling, including NSAIDs or mPGES-1 inhibitors, may be particularly valuable as an adjuvant strategy to target the TME and improve response to standard therapies. Identifying the most appropriate targets and drug combinations, while ensuring adequate brain and tumor exposure, will therefore be essential for translating these approaches to GBM treatment.

## 4 Platelets and glioblastoma

Emerging evidence underscores the TME’s critical role in glioma progression, highlighting its therapeutic relevance for aggressive brain tumors. Among the cellular components of the TME, platelets, traditionally known for their role in hemostasis, also play a crucial role in inflammation and cancer progression (Olsson and Cedervall, 2018). In GBM, platelets influence cancer behavior in various ways, including promoting angiogenesis, invasion, and chemotherapy resistance (Olsson and Cedervall, 2018; Haemmerle et al., 2018). Furthermore, platelet interactions with non-malignant cells within the TME, such as pericytes and immune cells, contribute significantly to tumor growth (Schleicher et al., 2015). While the prognostic significance of systemic platelet counts in glioma remains debated (Brockmann et al., 2007), recent studies indicate that elevated intratumoral platelet infiltration is associated with adverse outcomes in GBM. In contrast, lower perioperative platelet aggregation is associated with improved prognosis (Sloan et al., 2022). Although platelet-released cytokines stimulate GBM cell and endothelial proliferation *in vitro*, definitive *in vivo* confirmation of this direct growth promotion is still awaited. This suggests that increased platelet presence within the tumor mass might result from tumor-driven recruitment (Brockmann et al., 2011). Di Berardino et al. demonstrated that TXA_2_, the major product of the COX pathway in activated platelets, promotes the acquisition of a more aggressive phenotype in GBM cells by inducing COX-2 expression and activating molecular programs associated with EMT, accompanied by increased PGE_2_ biosynthesis (Di et al., 2026). The inhibition of platelet TXA_2_ synthesis with aspirin, as well as pharmacological blockade of the TXA_2_ receptor, attenuates the activation of COX-2/EMT-associated pathways, while selective COX-2 inhibition reduces PGE_2_ production and impairs GBM spheroid growth (Wang et al., 2011; Di et al., 2026) (Figure 2).

**FIGURE 2:**
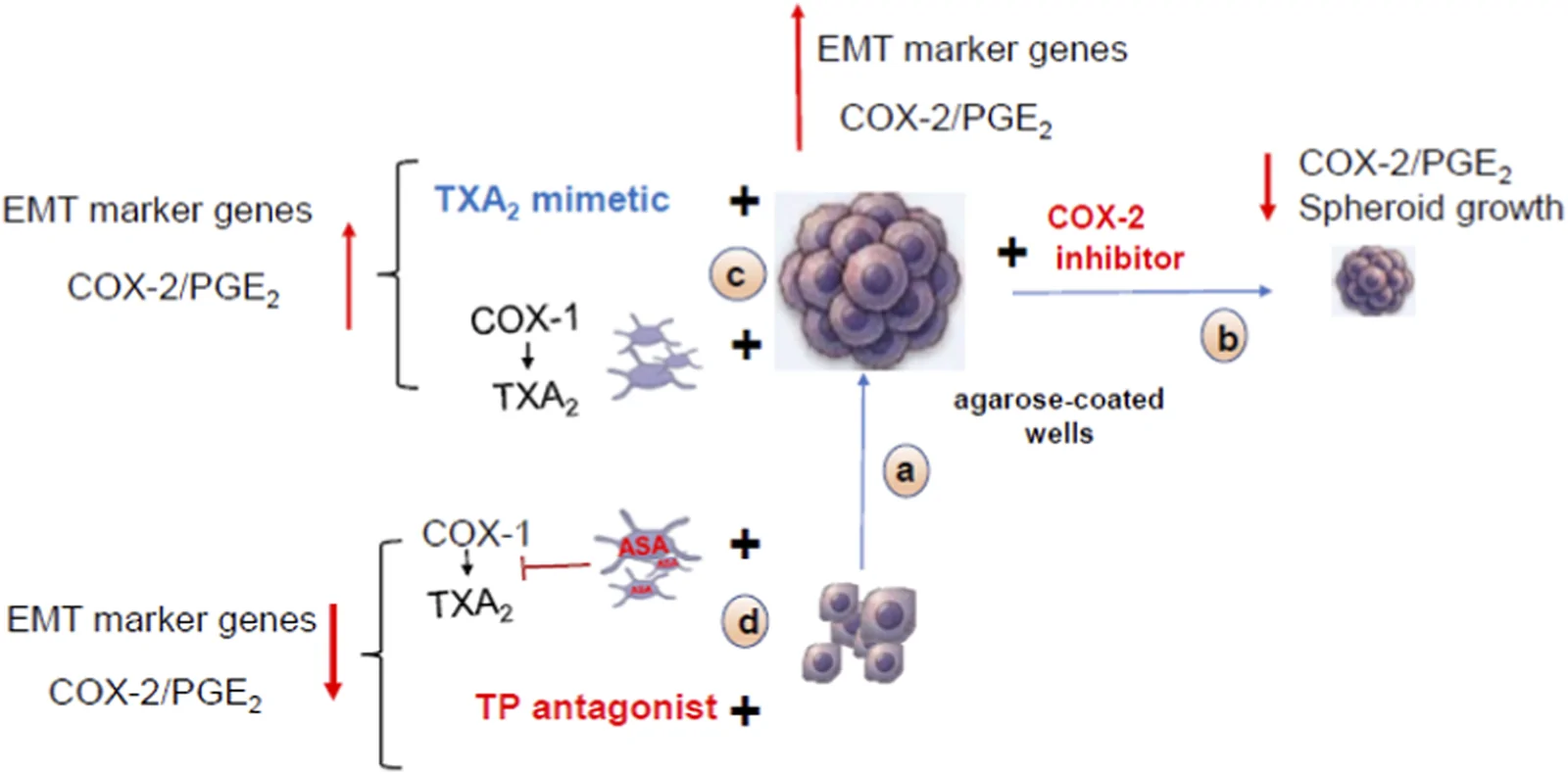
Platelet TXA_2_ induces the expression of COX-2 and enhances EMT marker genes in human U-87MG cells. **(a)** Human glioblastoma (GBM) U-87MG cells were dissociated into single cells, seeded into agarose-coated wells at approximately 1 × 10^5 cells, and incubated at 37 °C with 5% CO_2_ for up to 8 days; COX-2 expression and PGE_2_ biosynthesis increased during the formation of U-87MG GBM cell spheroids. **(b)** The selective COX-2 inhibitors celecoxib and rofecoxib inhibited PGE_2_ biosynthesis and reduced U-87MG spheroid growth. **(c)** A TXA_2_ mimetic or the addition of platelets to U-87MG spheroids, associated with enhanced TXA_2_ biosynthesis, enhanced COX-2/PGE_2_ and EMT marker genes. **(d)** In the co-culture with platelets selectively exposed to aspirin (ASA) to inhibit COX-1-dependent TXA_2_ generation, or in the presence of a TXA_2_ receptor (TP) antagonist, the expression of COX-2/PGE_2_ and EMT gene expression was restrained.

Furthermore, a significant bidirectional interaction between platelets and GSCs has been demonstrated, which contributes to local invasion and recurrence. Platelets tend to localize within GSC-rich niches, where tumor-derived thrombin activates them. This activation promotes GSC proliferation and preserves their stem-like characteristics (Sloan et al., 2022). The significant reduction in glioma growth confirms the therapeutic potential of targeting interactions between platelets and GSCs, as observed *in vivo* following pharmacological inhibition of Factor X and thrombin (Sloan et al., 2022). GSCs can also interact with platelets within the vascular compartment, promoting platelet activation, transforming growth factor-beta 1 (TGF-β1) secretion, and consequent suppression of NK cell antitumor activity (Wang et al., 2016). GSCs express higher levels of tissue factor than non-stem tumor cells, promoting local coagulation and platelet aggregation (Figure 3); this mechanism protects circulating or invasive GSCs from immune surveillance (Wang et al., 2016).

**FIGURE 3:**
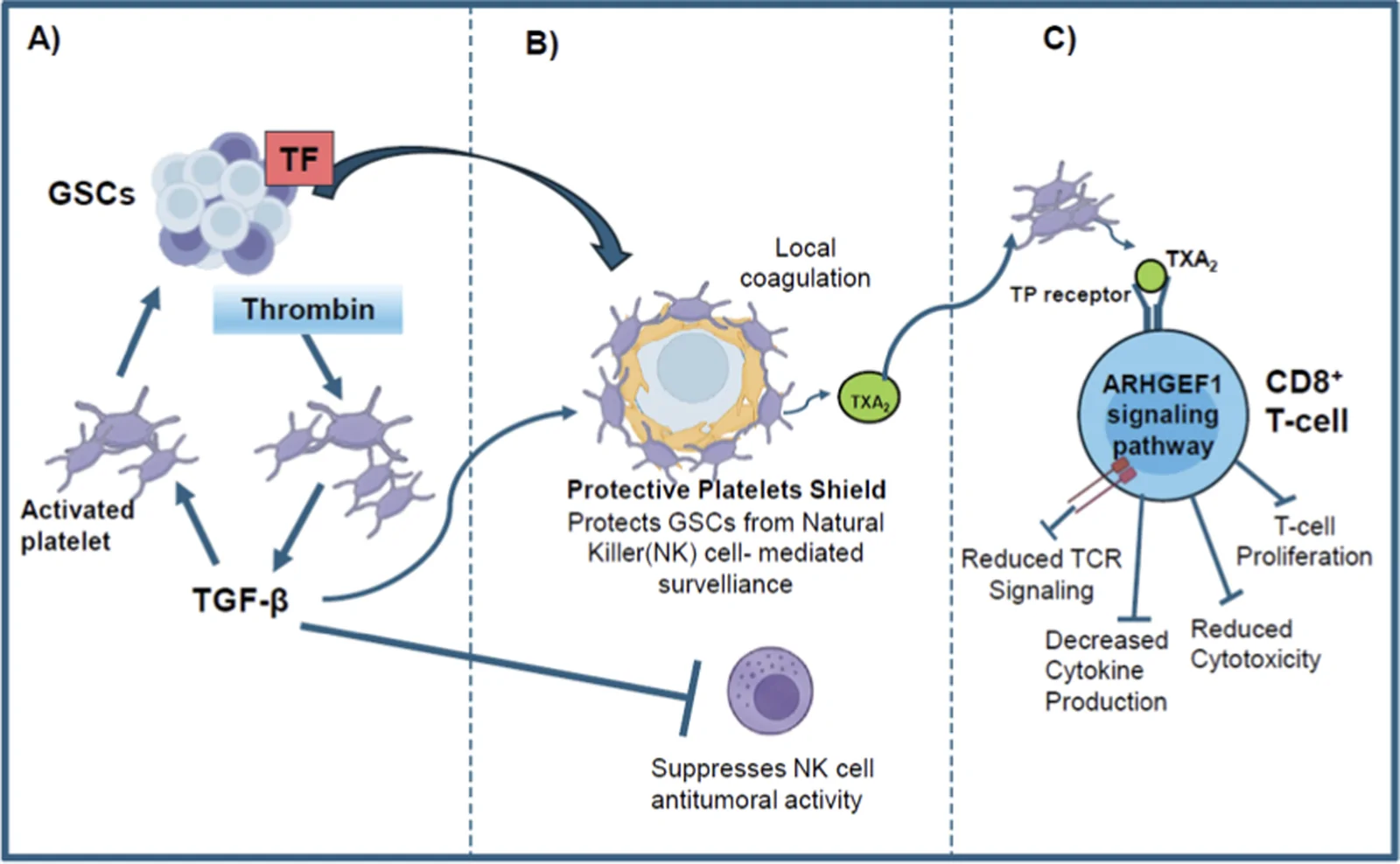
Platelet-Glioblastoma Crosstalk and TXA_2_- Mediated Immunosuppression: **(A)** GSC-platelet interaction: Glioma stem cells (GSCs) express tissue factor (TF) and secrete thrombin to recruit and activate platelets. In turn, activated platelets release TGF-β, driving GSC proliferation, stemness, invasion, and chemoresistance. **(B)** Immunosuppression and Coagulation: Local coagulation forms a protective platelet shield around GSCs, evading Natural Killer (NK) cell surveillance, while platelet-derived TGF-β suppresses NK cell activity. In addition, activated platelets release TXA_2_. **(C)** TXA_2_-Mediated CD^8+^ T-Cell Inhibition: TXA_2_ from platelets directly inhibits CD8^+^ T-cell function by activating the TP receptor and the ARHGEF1 signaling pathway. This leads to reduced T-cell receptor signaling, lower T-cell proliferation, and decreased cytokine production and cytotoxicity.

TMZ chemotherapy remains the frontline treatment for gliomas, yet therapeutic resistance routinely develops, frequently mediated by GSCs that drive tumor recurrence and poor patient survival (Dubrow et al., 2013). Platelets contribute substantially to this chemoresistant phenotype by secreting factors like TGF-β1 (Figure 3), which activate major pro-survival pathways in tumor cells, including the MEK/ERK and PI3K/AKT axes (Chen et al., 2013). Indeed, the platelet secretome contains numerous growth factors and cytokines such as EGF, PDGF, TGF-β, IGF, and CCL5 that enhance the stemness, viability, and chemoresistance of glioma cells (Casagrande et al., 2021). Therefore, targeting the molecular crosstalk between platelets and GSCs represents a promising strategy to overcome therapeutic resistance. Concurrently, advances in liquid biopsy technology have positioned blood components, including plasma, serum, EVs, and circulating cells, as invaluable tools for noninvasive cancer diagnosis and monitoring (Nikanjam et al., 2022).

Common blood-based biomarkers include cell-free DNA (cfDNA), circulating tumor DNA (ctDNA), and circulating tumor cells (CTCs) (Nikanjam et al., 2022). However, while ctDNA is highly detectable in many systemic cancers, its presence in the blood of glioma patients is severely limited, appearing in less than 10% of cases due to the BBB (Bettegowda et al., 2014); similarly, the detection efficiency of circulating glioma cells remains highly variable (Soffietti et al., 2022).

In this context, platelets, which are highly abundant and easy to isolate, have emerged as promising diagnostic and prognostic biomarkers in gliomas. Studies comparing platelet RNA profiles between glioma patients and healthy controls have demonstrated that the EGFRvIII mutation, a common genetic alteration in GBM, can be detected in the platelets of 80% of patients harboring it in their primary tumor (Nilsson et al., 2011). This phenomenon is attributed to platelets’ ability to sequester tumor-derived microvesicles that contain mutated nucleic acids. These tumor-educated platelets (TEPs) display distinct RNA splicing profiles that can accurately discriminate GBM patients from healthy individuals with 95% accuracy, and from patients with neuroinflammation or other non-malignant brain tumors with 80% accuracy in a minimally invasive manner (Sol et al., 2020). Furthermore, TEP RNA levels decrease significantly after surgical resection and rise again upon tumor recurrence, demonstrating their potential for monitoring longitudinal therapeutic response, with reported accuracy of 85% (Sol et al., 2020). Recently, combining RNA-sequenced TEPs with machine learning has shown promise for identifying biomarkers and developing personalized diagnostic strategies, including liquid biopsy-based cancer classification (Cygert et al., 2023). In conclusion, platelets significantly influence GBM progression and chemoresistance via intricate signaling loops, while tumor-educated platelets show important clinical promise as non-invasive biomarkers. Dissecting platelet–tumor interactions and translating them into liquid biopsy approaches may fundamentally improve glioma diagnosis, monitoring, and treatment.

### 4.1 Platelet TXA_2_ suppresses the immune response

When activated, platelets release a variety of bioactive substances that can significantly influence both innate and adaptive immune responses. This contributes to a highly immunosuppressive tumor environment (Chen L. et al., 2023; Labe et al., 2014). One of these substances is TXA_2_, a lipid derived from AA through COX-1 and thromboxane synthase (TBXAS1). TXA_2_ has been identified as a key regulator of immune cell function and the interaction between blood vessels and immune cells (Smyth et al., 2009). The immunomodulatory effects of TXA_2_ were first shown by Kabashima et al., who found that signaling through the TP receptor negatively affects dendritic cell–T-cell interactions, thereby reducing T-cell priming and clonal expansion (Kabashima et al., 2003); thus highlighting that TXA_2_ is involved not only in platelet aggregation and blood vessel tone but also in regulating adaptive immune responses (Smyth et al., 2009). Recently, Yang et al. (2025) discovered that TXA_2_ from platelets directly inhibits CD8^+^ T-cell function by activating the TP receptor and the ARHGEF1 signaling pathway. ARHGEF is a Rho guanine nucleotide exchange factor that links extracellular signals to RhoA GTPase activation. It functions downstream of the TP receptor/Gα12/13. This leads to reduced T-cell receptor signaling, lower proliferation, and decreased cytokine production and cytotoxicity (Yang et al., 2025) (Figure 3). Notably, blocking TXA_2_ production in platelets enhances T-cell-driven antitumor immunity and reduces metastasis. This underscores TXA_2_ as a crucial factor in platelet-driven immune suppression (Haemmerle et al., 2018; Yang et al., 2025). Although the specific role of the TXA_2_–TP pathway in GBM has not been fully explored, several disease characteristics suggest it may be important. GBM displays significant immunosuppression, including T-cell dysfunction and exhaustion, increased numbers of regulatory T cells, accumulation of tumor-associated macrophages, and MDSCs. It also shows increased levels of inhibitory cytokines, such as TGF-β and IL-10 (Woroniecka and Fecci, 2018).

Additionally, GBM patients often show systemic platelet activation and changes driven by platelets that correlate with poor outcomes and tumor development (Marx et al., 2018). In this context, TXA_2_ from platelets might aid immune evasion by hindering the activation and function of CD8^+^ T-cells that react to tumors and weakening the priming of dendritic cells and T-cells in lymphoid tissues. Furthermore, TXA_2_ is unlikely to work alone. It likely interacts with other platelet-derived substances that modulate the immune response, such as TGF-β, VEGF, platelet factor 4 (PF4), and platelet-derived extracellular vesicles (PEVs), all of which are linked to immune suppression and tumor growth (Labe et al., 2014; Xu et al., 2018). This coordinated signaling from platelets may be especially significant in GBM, where multiple overlapping immunosuppressive pathways collaborate to hinder effective antitumor immunity and reduce immunotherapy response rates (Lim et al., 2018). TXA_2_ may further promote T-cell dysfunction and immune escape. Targeting platelet-derived TXA_2_ therefore warrants investigation as a strategy to restore antitumor immunity and improve immunotherapy in GBM (Haemmerle et al., 2018; Yang et al., 2025).

## 5 Platelets as a drug delivery system

Platelets are anucleate cells derived from the cytoplasmic fragmentation of megakaryocytes, with a circulating half-life of approximately 8–10 days (Machlus and Italiano, 2013). In addition to their classical role in hemostasis and thrombosis, platelets also play a critical role in cancer. They can protect circulating tumor cells from mechanical stress and immune elimination, promote their adhesion to the vascular endothelium, reduce NK- and T-cell-mediated immunosurveillance, and facilitate the establishment of metastatic lesions (Haemmerle et al., 2018; Labe et al., 2014). This natural interaction between platelets and tumor cells has stimulated interest in their use as carriers for anticancer agents. Several characteristics make platelets promising candidates for cell-based drug delivery. Their relatively long lifespan in the bloodstream may prolong drug circulation and improve the pharmacokinetic profile of compounds that are usually administered intravenously. They can also be collected through minimally invasive procedures, manipulated *ex vivo,* and subsequently reinfused into the same patient, thanks to their low intrinsic immunogenicity. In addition, platelet surfaces display numerous binding sites that can be used for drug loading or functionalization, and they naturally express receptors and signaling pathways that enable recognition of damaged vasculature, activated endothelial cells, and tumor-associated signals. Platelets have also inspired cell-free delivery systems based on biomimetic platelets and PEVs, discussed in Section 5.3.

### 5.1 Drug-loaded platelets

One of the main platelet-based drug delivery strategies is directly loading therapeutic agents into intact platelets. Drug-loaded platelets have been widely explored preclinically for the delivery of small molecules (Zhao et al., 2023; Cacic et al., 2023; Xu et al., 2017; Lu et al., 2025; Dong et al., 2023; Wu et al., 2020; Tanak et al., 2022; Rao et al., 2018; Nishikawa et al., 2014; Abdelgawwad et al., 2018; Lv et al., 2021). The specific loading strategy depends mainly on the cargo’s physicochemical properties. The drug can be incorporated by passive diffusion, electroporation, endocytosis-mediated internalization, or membrane fusion. Passive diffusion is the simplest route, and Doxorubicin is by far the most extensively studied cargo in this category (Zhao et al., 2023; Xu et al., 2017; Lu et al., 2025; Dong et al., 2023; Wu et al., 2020). Typically, drug loading is achieved by incubating platelets with the therapeutic compound, and the drug enters the platelet along its concentration gradient, a process that the open canalicular system may facilitate. For storage feasibility, cryopreserved, clinical-grade doxorubicin-loaded platelets have been demonstrated to retain functionality and outperform liposomal doxorubicin formulations (Wu et al., 2020). Doxorubicin-loaded platelets have also been tested by decorating platelets with the glucose-functionalized glycolipid DSPE-PEG-Glucose to improve GLUT1-mediated tumor-cell binding and postoperative drug delivery (Zhao et al., 2023), or by conjugating an anti-CD22 antibody to the platelet surface to enhance lymphoma delivery (Xu et al., 2017), or by conjugating liposomes containing quercetin (Lu et al., 2025). The same passive-loading principle has been extended to tyrosine-kinase inhibitors (sorafenib, lenvatinib). It has shown enhanced tumor uptake in a rat model of hepatocellular carcinoma compared with free drug (Tanak et al., 2022). Electroporation is a suitable alternative for loading cargo that cannot passively cross membranes. A representative example is platelets electroporated to incorporate gold nanorods (a photothermal agent) in a mouse model of head and neck squamous cell carcinoma, where platelet-mediated delivery enhanced tumor accumulation of the photothermal agent and significantly improved the antitumor efficacy of laser-induced photothermal therapy (Rao et al., 2018). Moreover, platelets possess a natural “scavenger” function, taking up small molecules, nucleic acids, and particulate material without degrading them, and have therefore been described as “covercytes” rather than phagocytes (Banerjee et al., 2020; Gaertner et al., 2017; White, 2005). The intrinsic ability of platelets to incorporate and transport particulate material has also been exploited to load inactivated Sendai virus envelopes. After systemic administration in melanoma-bearing mice, the resulting platelet-virus complexes preferentially accumulated in tumor tissue, promoted immune-cell recruitment, and significantly suppressed tumor growth (Nishikawa et al., 2014). Similarly, the tumor-suppressive cytokine CXCL10 was loaded via *ex vivo* incubation and released from reinfused platelets within the TME, promoting antitumor immunity in a melanoma model (Li Y. T. et al., 2016). Similarly, receptor-mediated internalization has been used to load platelets with biotinylated nanoparticles co-encapsulating a photothermal agent and the immunostimulant imiquimod, achieving potent antitumor efficacy in multiple preclinical models, including patient-derived xenografts and humanized mice (Lv et al., 2021). Membrane fusion represents an alternative one-step engineering strategy. Fusion of platelets with IL-15-functionalized, doxorubicin-loaded liposomes achieved loading efficiencies exceeding 70%, enabling simultaneous cytoplasmic drug loading and surface cytokine modification while preserving platelet targeting properties. The engineered platelets effectively suppressed lung metastasis and postoperative tumor recurrence in murine melanoma models (Zhao et al., 2024).

Platelets can also be loaded by genetically engineering their precursor megakaryocytes (MKs) or hematopoietic progenitor cells (HSPCs). Zhang X. et al. (2018) engineered platelets from murine MKs to express the programmed cell death protein 1, PD-1, and loaded them with a regulatory T-cell (Treg) inhibitor (Scurr et al., 2017). The resulting platelets were administered in a melanoma mouse model subjected to partial surgery, where they accumulated within the TME, blocked PD-L1, inhibited Treg activity, and enhanced CD8^+^ T-cell response, significantly delaying tumor progression and prolonging survival (Scurr et al., 2017). Similarly, murine HSPCs were genetically engineered to generate platelets expressing surface-bound tumor necrosis factor-related apoptosis-inducing ligand (TRAIL), which selectively induced tumor-cell apoptosis and significantly reduced metastasis in a mouse model of prostate cancer (Li J. et al., 2016).

### 5.2 Drug-conjugated platelets

Platelets can also be modified directly through bioconjugation techniques that link small molecules, antibodies, nanoparticles, or other therapeutics directly to their surface by exploiting the abundance of binding sites available on the plasma membrane. In recent years, several covalent and non-covalent conjugation strategies have been tested. One example of covalent conjugation exploits platelet surface glycoproteins enriched in sialic acid residues to form reversible covalent bonds with boronic acid-modified molecules; this approach was tested to attach nanogels to the platelet surface while preserving tumor-targeting behavior in a melanoma model (Yan et al., 2022). Moreover, primary amine groups on platelet membrane proteins can react with N-hydroxysuccinimide (NHS) or sulfo-NHS esters, enabling platelet surface functionalization without disrupting platelet-cancer cell interactions. This strategy has been exploited to conjugate imaging probes, targeting ligands, and enzymes onto intact platelets. For example, Zhang et al. functionalized platelets with an NHS-linked near-infrared probe to generate multifunctional platelet vehicles for image-guided chemo-photothermal therapy (Zhang et al., 2022). Using the same chemistry, Tang et al. introduced urease onto the platelet surface. Asymmetric urease immobilization generated Janus platelet micromotors capable of urea-powered propulsion. Platelets were loaded with doxorubicin and retained their ability to interact with breast cancer cells, increasing contact frequency with target cells and enhancing the therapeutic activity of the transported cargo (Tang et al., 2020). The most widely used covalent strategy, however, is maleimide–thiol coupling, usually used to link immune-checkpoint inhibitors (ICIs) to the platelet surface. This approach has been demonstrated to facilitate targeted delivery of ICIs to residual cancer cells in post-surgical cavities in melanoma and breast cancer models, effectively inhibiting tumor regrowth (Wang et al., 2017b; Wang et al., 2023; Hu et al., 2021; Lu et al., 2022). The growing body of preclinical work on ICI-conjugated platelets has led to a Phase 1 clinical trial evaluating the safety and tolerability of IL-2-conjugated platelets, given alone or in combination with anti-PD-1, in patients with advanced solid tumors (NCT05829057). A further covalent route consists of introducing azide groups onto the platelet membrane to enable catalyst-free click chemistry with dibenzocyclooctyne. This approach was used to conjugate nanogels containing a pore-forming peptide and granzyme B (Fan et al., 2022) or to conjugate platelets to other cell types (Hu et al., 2018). For example, platelets decorated with anti-PD-1 antibodies have been conjugated to hematopoietic stem cells (HSCs) and have suppressed leukemia growth and recurrence in mice (Hu et al., 2018). Non-covalent conjugation avoids irreversible chemical modification of platelet surface proteins, and it relies on the platelet’s lipid bilayer, electrostatic surface properties, and receptor presentation. For example, zwitterionic phospholipid head groups can mediate electrostatic attachment of anionic cargo (Yang et al., 2023) or a negative surface favors electrostatic adsorption of positively charged cargo (Gao et al., 2023). Specific platelet surface receptors, such as integrin αIIbβ3, can also serve as non-covalent anchors for cargo attachment (Hansen et al., 2017).

### 5.3 Beyond whole-platelet carriers: Biomimetic platelets and PEVs

Conventional anticancer treatments are frequently limited by poor tumor selectivity, unfavorable pharmacokinetics, dose-limiting toxicity, and insufficient tumor penetration. These limitations have stimulated interest in cell-derived carriers, including biomimetic platelets and PEVs. Biomimetic platelets consist of synthetic nanoparticles (NPs) coated with biological platelet membranes, which improve circulation time, immune evasion, and tumor targeting compared with uncoated nanoparticles (Shi et al., 2017; Bose et al., 2018; Wu et al., 2019; Hu et al., 2015). Platelet membranes, typically isolated from platelet-rich plasma, are transferred onto nanoparticles by extrusion, sonication, or microfluidic approaches, conferring platelet-like biological properties that enhance accumulation within tumor tissue (Fang et al., 2023). The first landmark study demonstrating the feasibility of this approach used TRAIL-functionalized platelet membranes to coat doxorubicin-loaded NPs. The specificity of the interaction between platelet P-selectin and CD44 expressed on tumor cells enhanced tumor accumulation and markedly suppressed tumor growth and metastasis in mice bearing MDA-MB-231 breast tumors (Hu et al., 2015). A growing number of studies have tested this approach for cancer therapy, and coating cytotoxic-agent-loaded NPs with platelet membranes is the most widely adopted approach. Formulations include polymeric particles encapsulating bufalin (Wang et al., 2019), doxorubicin (Hu et al., 2015), or bortezomib (Hu et al., 2015), and paclitaxel nanocrystals (Mei et al., 2020), all showing improved tumor accumulation and reduced off-target toxicity. Platelet-membrane coating has also been used to potentiate radiotherapy effects (Chen et al., 2019) and, more recently, to improve ferroptosis-based immunotherapy using PD-1-combined, platelet-membrane-camouflaged magnetic nanoparticles loaded with sulfasalazine (Jiang et al., 2020). Importantly, the translational potential of this strategy is demonstrated by CE120, a platelet membrane-coated polymeric nanoparticle loaded with the TLR agonist resiquimod, which received U.S. FDA Investigational New Drug (IND) clearance in 2023. Preclinical studies using this type of platelet membrane-coated NP have demonstrated durable antitumor immunity and inhibition of tumor progression in murine colorectal and breast cancer models (Bahmani et al., 2021).

In recent years, EVs have emerged as promising biological carriers for therapeutic delivery owing to their intrinsic biocompatibility, ability to transport a broad spectrum of biomolecules, and capacity to mediate intercellular communication. As the most abundant EV population in circulation under physiological conditions (Puhm et al., 2021), PEVs have attracted increasing attention because they combine many advantages of biological EVs with unique properties inherited from platelets. PEV membrane is enriched in platelet adhesion molecules, receptors, and bioactive lipids that facilitate interactions with recipient cells. At the same time, the phospholipid bilayer protects therapeutic cargo from degradation and may improve circulation stability. Their nanoscale size also facilitates tissue penetration beyond that of intact platelets, and platelet concentrates provide a clinically established and abundant source for their production. However, PEV-based drug delivery remains at an early preclinical stage, with relatively limited supporting evidence (Wu et al., 2023).

Two main strategies can generate PEV-based delivery systems. Therapeutic agents can be loaded into parental platelets before vesicle generation, or into isolated PEVs after their production by passive incubation or membrane-disruptive methods. PEVs can encapsulate both hydrophilic and hydrophobic drugs (Soleymani et al., 2019). By acquiring CD47 from their parent platelets, they exploit the “do not eat me” signal to evade immune clearance *in vivo* (Gamonet et al., 2020). PEVs transfer their cargo to malignant cells through direct fusion or endocytosis, improving intracellular drug uptake. For example, paclitaxel-loaded PEVs inhibited breast cancer cell migration and invasion *in vitro* while showing limited toxicity to non-malignant cells (Meliciano et al., 2023). Compared with free doxorubicin, doxorubicin-loaded PEVs showed greater accumulation in leukemia cells (Kailashiya et al., 2019) and greater cytotoxicity against breast, colon, and lung cancer cells, with reduced systemic drug release (Wu et al., 2020). PEVs can also acquire macromolecular cargo via membrane fusion with drug-loaded liposomes. Fusing PEVs with liposomes encapsulating the enzyme chloroperoxidase enabled efficient loading of this macromolecule while preserving the intrinsic tumor-targeting capability of PEVs in a murine breast cancer model, with potential for co-delivering small molecules and macromolecules for combination therapy (Ning et al., 2023).

However, clinical translation of PEVs remains limited by challenges in standardized production, batch-to-batch consistency, and scalable manufacturing while preserving vesicle integrity, despite their favorable tissue tropism and immune-evasion properties.

Johnson et al. (2023) reported the first-in-human, randomized, double-blind, placebo-controlled Phase I trial of clinical-grade platelet EVs, demonstrating that they were safe and well tolerated in healthy volunteers. This study provides an important proof of clinical feasibility for platelet EV-based therapeutics. Table 2 summarizes the main studies that have investigated PEVs as carriers for anticancer therapeutics.

**TABLE 2:** Key studies on the use of platelet-derived vesicles as drug delivery systems.

Loading strategy	Cargo	PEV release	Model	References
Passive incubation of platelets with the drug	Doxorubicin	Activation of platelets with ADP	HeLa and Jurkat cell lines	(Sarkar and Dasgupta, 2015)
Passive incubation of platelets with drug-loaded nanoparticle	Doxorubicin-loaded nanoparticles	Activation of platelets with ADP	HeLa and Jurkat cell lines	(Kailashiya et al., 2019)
Passive incubation of platelets with the drug	Doxorubicin	Activation of platelets with A23187	Human leukemia cell lines (HL 60 and K562) and HUVECs	(Kailashiya et al., 2019)
Direct incubation of PEVs with drug	Doxorubicin	Extrusion/sonication/freeze-thaw/incubation	Embryonic fibroblast cell line and Triple-negative breast cancer cell lines	(Wu et al., 2023)
Direct incubation of PEVs with drug	Paclitaxel	PEVs naturally released during the storage of clinically expired platelet concentrates	breast cancer and endothelial cells	(Meliciano et al., 2023)

### 5.4 Mechanisms of drug release

While considerable progress has been made in loading therapeutic agents into platelets and platelet-derived carriers, less attention has been devoted to controlling when and where these agents are released. Indeed, cargo release is a critical aspect of any drug delivery system, since it directly determines specificity, kinetics, and the risk of premature (“off-target”) drug release. The earliest proof-of-concept studies mainly focused on demonstrating the feasibility of using platelets as drug carriers. In these systems, cytotoxic agents were loaded into platelets and released largely through passive mechanisms, with limited control over the timing or location of drug release. More recently, research has shifted toward engineering carriers that release their cargo only after reaching the tumor. This strategy exploits both the intrinsic tumor-homing properties of platelets and the unique biochemical features of the TME. In particular, platelets and platelet-derived systems (biomimetic platelets and PEVs) are designed as stimuli-responsive systems that release their cargo in response to local tumor-associated signals, such as acidic extracellular pH, elevated hydrogen peroxide (H_2_O_2_), or high intracellular glutathione levels. By coupling drug release to these tumor-specific signals, these platforms aim to maximize drug delivery at the target site while minimizing systemic exposure and unwanted toxicity. Table 3 summarizes the main release mechanisms currently used in platelet-based delivery systems.

**TABLE 3:** Overview of the main release mechanisms used in platelet-based drug delivery systems.

Mechanism	How it works	Key evidence	Spatial specificity	Risks
Passive, diffusion-driven release	release is concentration-gradient-driven leakage, with no dedicated molecular switch	(Sarkar et al., 2013)	Low	Premature systemic leakage; reduced tumor drug levels
Activation-dependent release	drug is released as a consequence of platelet activation by a defined agonist (thrombin, collagen, ADP), the same stimuli that trigger native secretion	(Wang et al., 2017b)	Moderate. Depends on local thrombin/agonist activity	Unintended systemic platelet activation; thrombotic risk
pH-dependent release	Exploits the characteristically acidic tumor microenvironment as a release trigger via pH-responsive loading/linker chemistry	(Xu et al., 2017)	Moderate–high/exploits a consistent tumor feature	Incomplete release in less acidic tumor regions
Redox-dependent release	Exploits elevated tumor-microenvironment H_2_O_2_ and intracellular glutathione (GSH) via redox-cleavable linkers	(Jiang et al., 2020)	Moderate–high	Variable efficacy across tumor types; oxidative-stress side effects
Externally triggered (physical) release	Uses externally controlled triggers (light or heat) rather than tumor-intrinsic cues	(Chen et al., 2019; Chan et al., 2015; Jing et al., 2018)	High at the irradiated/targeted site; none elsewhere	Limited to superficial or imageable lesions
Multi-stimulus/sequential-release systems	Combines multiple release mechanisms for sequential, rather than simultaneous, payload delivery	(Zhang X. et al., 2018; Scurr et al., 2017)	Potentially highest	Most complex to manufacture, validate, and optimize

### 5.5 Translational considerations for platelet-based drug delivery in brain tumors

Although platelet-based drug delivery platforms have shown promising potential in preclinical studies, their application to primary brain tumors remains unexplored. Most of the studies discussed so far have been carried out in non-CNS tumor models, including breast cancer, melanoma, lymphoma, colorectal cancer, and other solid malignancies, and should therefore be considered proof-of-concept evidence supporting the feasibility of platelets and platelet-derived systems as tumor-targeted carriers, not as direct evidence of efficacy in the brain. Translation of these findings to GBM requires particular caution because brain tumors present a unique pharmacological obstacle: the BBB and its tumor-associated counterpart, the blood-tumor barrier (BTB). The BBB is formed by specialized brain microvascular endothelial cells connected by tight junctions and supported by pericytes, astrocytic end-feet, and other components of the neurovascular unit. It is a highly selective interface that protects the CNS and strongly limits the intracerebral accumulation of many therapeutic compounds (Arvanitis et al., 2020). In GBM, the BBB and BTB are highly heterogeneous: angiogenic remodeling and abnormal vascular architecture increase permeability in highly vascularized regions, whereas in other areas of the tumor, the BBB may remain functional (Sarkaria et al., 2017).

An effective drug delivery strategy for GBM should therefore reach the tumor and overcome the BBB/BTB to achieve adequate distribution throughout the tumor tissue. Overcoming these barriers is therefore a critical consideration for translating platelet-based delivery systems to GBM. It may differ substantially depending on the nature and size of the carrier used. Intact platelets, platelet membrane-coated nanoparticles, and PEVs may indeed rely on different mechanisms to reach the brain. For intact platelets, efficient BBB crossing should not be assumed. Although intact platelets have been documented in GBM tissue (Sloan et al., 2022) and whole platelets can migrate across a compromised BBB under specific pathological signaling (Chen et al., 2026), it remains unclear whether their spontaneous intratumoral accumulation is sufficient to achieve therapeutically relevant drug concentrations. The potential value of platelets as a drug delivery system in brain tumors may instead derive from their intrinsic ability to interact with activated/damaged endothelium and tumor-associated vessels, where platelet activation could promote local release of the encapsulated or surface-conjugated therapeutic cargo. Once released, the drug must cross the BBB or the BTB, unless it is released directly inside tumor vessels that are already permeable enough.

Platelet membrane-coated nanoparticles may offer an advantage over intact platelets because their nanoscale size can facilitate tissue penetration. However, their reduced dimensions do not necessarily ensure efficient BBB crossing, particularly in GBM regions where the barrier remains relatively intact. Moreover, evidence supporting the use of platelet membrane-coated nanoparticles in brain tumors remains limited, as most studies have been conducted in non-CNS tumor models.

PEVs may represent an attractive intermediate strategy because they combine platelet-derived membrane components with nanoscale dimensions and the ability to transport proteins, lipids, nucleic acids, and pharmacological agents. Although EVs from different cellular sources have been shown to cross the BBB, and under conditions in which BBB permeability increases, PEVs likely have greater access to the CNS, either via enhanced transcytosis or paracellular leak, whether PEVs actually cross the BBB remains poorly established (Puhm et al., 2021; Yeh et al., 2025). However, a limited intrinsic ability of a drug-delivery system to cross the BBB does not necessarily preclude its application to brain tumors, since various routes of administration and complementary strategies are currently available or under investigation to facilitate drug release into the central nervous system or bypass the barrier entirely (Nance et al., 2021; Narsinh et al., 2024). Systemic intravenous administration is the most straightforward route, but it requires the carrier to reach the cerebral vasculature and then overcome the BBB/BTB. EVs from different cellular sources have been shown to enter the CNS following vascular administration; however, brain delivery of unmodified EVs is generally inefficient, with less than 1% reported to reach the CNS, and appears to be strongly influenced by the cellular origin of the vesicles (Zhu et al., 2019; Guo et al., 2022). Moreover, systemic administration results in substantial peripheral distribution and clearance, particularly by the liver and spleen (Kang et al., 2021; Nieland et al., 2023). Nevertheless, several preclinical studies have demonstrated that EV engineering can enhance brain and tumor targeting (Choi et al., 2021), including in experimental models of glioma and GBM (Zhu et al., 2019; Guo et al., 2022). Intranasal administration represents an alternative non-invasive approach that can partially bypass the BBB through nose-to-brain transport pathways. Notably, engineered EVs administered intranasally have been shown to reach intracranial tumors and deliver therapeutic cargo in orthotopic mouse models of GBM (Wang K. et al., 2021). Although these findings provide important proof of concept for EV-based brain delivery, their applicability to PEVs remains to be established. Beyond alternative administration routes, BBB permeability can also be transiently increased; among these strategies, focused ultrasound allows localized, reversible BBB opening and has progressed to early clinical investigation in patients with glioma and GBM (Narsinh et al., 2024; Zhu et al., 2024). Such approaches could improve CNS access of platelet-derived nanoparticles or PEVs, although this possibility remains largely unexplored (Kline-Schoder et al., 2025; Del Bene et al., 2026). Alternatively, local delivery strategies can bypass the BBB altogether. Convection-enhanced delivery (CED), for example, enables direct administration of therapeutic agents into the brain parenchyma, allowing high local concentrations while reducing systemic exposure (Rolfe et al., 2024). Although its combination with platelet-derived carriers has not been extensively investigated, local delivery could provide an alternative strategy for platelet-based nanoparticles or PEVs without requiring their intrinsic ability to cross the BBB. These approaches demonstrate that platelet-derived delivery systems could be integrated with technologies that temporarily enhance barrier permeability or with local administration strategies that bypass the BBB altogether. Overall, platelet-based drug delivery is a promising strategy, but its application to GBM remains in the early stages. Evidence from extracranial tumors provides strong proof of concept, while effective delivery across the BBB/BTB remains to be established. Future studies should therefore define the brain and tumor distribution of these platforms and explore their potential integration with strategies that facilitate or bypass BBB crossing.

## 6 Conclusion

Addressing challenges such as improving early detection, promoting innovative treatments, and overcoming chemoresistance is essential for increasing survival rates in brain cancer patients. Improving existing tumor classification systems—primarily based on microscopic morphology and immunohistochemistry—by incorporating genomic data can markedly improve diagnostic accuracy. However, intratumoral heterogeneity remains a significant obstacle. Efforts should focus on using liquid biopsies and advanced imaging techniques to monitor and evaluate early tumor responses during treatment. In this context, emerging biomarkers, such as the omics profiles of TEPs, show great potential as non-invasive tools. Integrating RNA-sequenced TEPs with machine learning shows promise for developing future biomarkers and personalized diagnostics, such as liquid biopsies for cancer classification (Cygert et al., 2023).

Eicosanoids generated from AA by COX, LOX, and CYP450 enzymes are lipid mediators that participate in signal transduction pathways regulating neurotransmission, neuroinflammation, neuroplasticity, tumor growth, and immune evasion. Brain tumors can produce different eicosanoid families through cell-specific biosynthesis by cancer cells, microglia, infiltrating immune cells, and platelets. The eicosanoids generated in the tumor can influence tumor heterogeneity. A deeper understanding of how eicosanoids in the TME influence brain cancer phenotypes may reveal new therapeutic targets. We have discussed how investigating less-characterized cell populations within the cancer microenvironment, notably platelets, which can accumulate and release factors such as TXA_2_, might offer valuable insights into novel therapeutic strategies. Given TXA2’s role in immune regulation, exploring antiplatelet agents to support immune responses in brain cancer could be important. This strategy could advance immune therapies, including vaccines and cellular treatments like Chimeric Antigen Receptor (CAR) T-cells.

Progress in developing more efficacious therapies for brain tumors has been limited by inadequate preclinical models, including *in vitro* systems and subcutaneous xenograft models. Furthermore, they often do not provide a comprehensive assessment of critical features, such as the BBB. We have discussed innovative approaches that use platelets for targeted drug delivery—utilizing their capacity to transport therapeutics directly to tumor sites. This approach warrants further investigation to establish clearer evidence of its effectiveness in delivering drugs to brain tumors.

Looking ahead, the next decade in brain cancer treatment will likely be defined by these main paradigms: precision medicine using molecular and epigenomic profiling; improved drug delivery to cross the BBB; liquid biopsy that integrates platelet-based omics analysis with machine learning; enhancing antitumor immune response through combination immunotherapy and cellular strategies with drugs affecting platelet TXA_2_ biosynthesis; and targeting tumor-microenvironment interactions by repurposing drugs inhibiting eicosanoid biosynthesis. Improving treatment and survival of brain cancer patients requires not only scientific innovation, but also continuous investment in collaborative clinical trial infrastructure, equitable access to innovative therapies, and the routine incorporation of translational endpoints into clinical research. Only through such a coordinated, multidisciplinary strategy can the trajectory of brain cancer outcomes be effectively transformed.
